# Seeing without a Scene: Neurological Observations on the Origin and Function of the Dorsal Visual Stream

**DOI:** 10.3390/jintelligence12050050

**Published:** 2024-05-11

**Authors:** Robert D. Rafal

**Affiliations:** Department of Psychological and Brain Sciences, University of Delaware, Newark, DE 19716, USA; rrafal@udel.edu

**Keywords:** superior colliculus, parietal lobe, Bálint’s syndrome, hemispatial neglect, progressive supranuclear palsy

## Abstract

In all vertebrates, visual signals from each visual field project to the opposite midbrain tectum (called the superior colliculus in mammals). The tectum/colliculus computes visual salience to select targets for context-contingent visually guided behavior: a frog will orient toward a small, moving stimulus (insect prey) but away from a large, looming stimulus (a predator). In mammals, visual signals competing for behavioral salience are also transmitted to the visual cortex, where they are integrated with collicular signals and then projected via the dorsal visual stream to the parietal and frontal cortices. To control visually guided behavior, visual signals must be encoded in body-centered (egocentric) coordinates, and so visual signals must be integrated with information encoding eye position in the orbit—where the individual is looking. Eye position information is derived from copies of eye movement signals transmitted from the colliculus to the frontal and parietal cortices. In the intraparietal cortex of the dorsal stream, eye movement signals from the colliculus are used to predict the sensory consequences of action. These eye position signals are integrated with retinotopic visual signals to generate scaffolding for a visual scene that contains goal-relevant objects that are seen to have spatial relationships with each other and with the observer. Patients with degeneration of the superior colliculus, although they can see, behave as though they are blind. Bilateral damage to the intraparietal cortex of the dorsal stream causes the visual scene to disappear, leaving awareness of only one object that is lost in space. This tutorial considers what we have learned from patients with damage to the colliculus, or to the intraparietal cortex, about how the phylogenetically older midbrain and the newer mammalian dorsal cortical visual stream jointly coordinate the experience of a spatially and temporally coherent visual scene.

## 1. Introduction

    I am a part of all that I have met;

  Yet all experience is an arch wherethro’

Gleams that untravell’d world whose margin fades

   Forever and forever when I move.

        Ulysses

     Alfred, Lord Tennyson

In *The Wizard of Oz*, Dorothy asks the scarecrow, “What would you do with a brain if you had one”? Tennyson’s Ulysses answers that he would move. The ability to move enabled an explosion of opportunities for evolving new species to expand their niches and genetic variability and thereby to adapt flexibly to a changing environment. As the size of organisms increased, affording competative advantages, the sizes of their populations necesssarily decreased, and survival of species became increasingly dependent upon the survival of each of their individual organisms.

Every movement entails both risks and potential rewards for an organism. This reality requires that its movements be context-dependent. For a frog, for example, survival requires the abilility to quickly orient toward a small, moving object in its visual periphery (insect prey) and away from a large, looming object (a predator). This need for the ability to make context-dependent movements selected for the evolution of the nervous system. Göthe’s Faust tells us that, “In the beginning there was the act”. Organisms that do not move do not need to see and do not need a nervous system. The sea squirt, for example, moves like a tadpole in its larval stage under the control of its nervous system, but since sustaining a nervous system is intensely energy-demanding (the human brain constitutes 2% of our body weight but utilizes 20% of energy produced), the sea squirt eats its brain when it attains its sessile adult form.

To utilize visual information to guide motor behavior, it must be encoded in the reference frame of the body, that is, in egocentric coordinates. For example, to move your hand to grasp an object, you need to have information about where your hand is (derived from sensory information from your muscles, called proprioception—the sense of the position of your body parts relative to other body parts) and also where your eyes are looking.

As we will see, information about where your eyes are looking at any single instant is derived from copies of the motor commands that generate each eye movement. These eye movement commands are sent from the superior colliculus in the midbrain to the intraparietal cortex via the frontal eye field. The intraparietal cotex and the frontal eye field both receive visual information from the primary visual cortex via projections referred to as the dorsal visual stream. Visual information, and information about eye position in the orbit, are integrated in the intraparietal cortex to encode the locations of objects in egocentric coordinates.

This tutorial considers, from the perspective of a clinical neurologist, the origin and function of the dorsal visual stream—a function that enables us to experience, and to act in, the untravelled world that continuously fades as we move. In this tutorial, I will share observations from the clinic and from the laboratory of patients who suffered from degeneration of the superior colliculus and patients who sustained bilateral or unilateral injuries to the parietal lobe.

## 2. The Evolution of the Dorsal Visual Stream

All vertebrates transmit visual signals from the retina of the eye to the midbrain optic tectum (called the superior colliculus in mammals). The colliculus computes a salience map of locations and priorizes the most behaviorally salient locations as targets for action, i.e., an orienting response (with the eyes and/or body) toward (or away from) objects at the selected location. This mechanism provides for fast, and context-dependent, reflexive orienting; a frog, for example, will reflexively turn toward a small, moving object in its visual periphery (insect prey) and away from a large, moving stimulus (predator).

In mammals, a second anatomical pathway transmits visual signals from the retina to the primary visual (striate) cortex via the lateral geniculate nucleus of the thalamus; additionally, in predatory mammals including primates, the evolution of forward-facing eyes enabled binocular vision with more precise depth perception. This visual pathway to the visual cortex transmits two different kinds of visual signals from a duplex retina.

One information channel to the visual cortex that enabled efficient arboreal foraging in primates originates in the central fovea of the eye’s retina and transmits visual signals enabling high-resolution color vision and depth preception. This channel of information is transmitted via the parvocellular (small-cell) layers of the lateral geniculate nucleus of the thalamus to the primary visual cortex. From the primary visual cortex, this channel of visual information projects to the ventral extrastriate visual cortex on the undersurface of the occipital and temporal lobes. The projections from the primary visual cortex to the the ventral surfaces of the occipital and temporal lobes is referred to as the ventral visual stream.

A second visual information channel that transmits low-resolution information from across the entire retina is integrated with input from the superior colliculus to generate a visual salience map in the primary visual cortex (Li 2016). This visual channel is transmitted from the magnocellular (large-cell) layers of the lateral geniculate nucleus of the thalamus to the primary visual cortex, and from there, it projects to the extrastriate visual cortex on the dorsal surfaces of the parietal, temporal, and frontal lobes. This projection from the primary visual cortex to the dorsal cortical areas is referred to as the dorsal visual stream. The neural signals transmitted through the dorsal visual stream are utilized to regulate the orienting responses of the eyes, head, and body. In primates, information transmitted via the dorsal visual stream is also used to control manual movements of the hands; these movements can grasp and manipulate objects and can be positioned by shoulders that move in all three planes.

Thus, primates (including humans) have two image-forming visual pathways that are integrated to control orienting movements: a subcortical route via the retinotectal pathway from the retina to the superior colliculus that mediates fast, reflexive orienting responses to external (exogenous) stimuli (e.g., automatically turning toward—or away from—a sudden change in luminance or movement in the visual periphery) and a phylogenetically newer route via magnocellular projections from the lateral geniculate nucleus to the primary visual cortex that projects to the extrastriate cortex via the dorsal visual stream. Signals transmitted via the dorsal visual stream are utilized to select objects for action and for guiding voluntary (endogenous) orienting responses based on internal goals (e.g., looking both ways before crossing the street).

As noted above, acting in a changing environment that contains moving objects and changes continuously as we move requires that objects in the scene be encoded in egocentric coordinates (that is, in terms of their spatial relationships to our own body). Visual signals transmitted via the dorsal stream are used to reconstruct a representation of a visual scene in body-centered coordinates—a representation that encodes the spatial relationships between objects and our body and other objects. This information is used to control visually guided behavior and, as we will see, to select objects in the visual scene for access to conscious awareness.

How are these two channels of visual information integrated and co-ordinated to provide coherent experience and behavior? And how are the competing demands of changes in the external envinronment (a movement seen out of the corner of the eye) and the demands of the current aims of voluntary actions arbitrated and resolved? This tutorial takes a crack at addressing these questions from the perspective of a clinical neurologist.

It begins, as it must for a neurologist, at the bedside, considering the plight of patients with damage to the superior colliculus and patients with lesions of the dorsal visual stream. This tutorial then takes a look under the hood to explore how visual inputs to phylogenetically older and newer pathways are integrated to reconstruct a unified and continuously updated representation of a visual scene that is used in contolling visually guided actions.

The scenes that we experience seeing, however, are much richer than the limited number of objects that we may select to act on at any one instant. This tutorial ends by asking the question, “what is a scene”? And it considers how what we see is a representation that integrates information from both the ventral and dorsal streams.

## 3. Interpretating Neurological Observations: First Principles

The behaviors we observe in victims of brain injury can be preplexing and, at times, may even seem bizaare. Making sense of such behaviors requires an appreciation of the biological and evolutionary constraints on how behavior can be influenced by brain injury and its compensatory reorganization. As we proceed, it will help to keep some basic principles in mind:

- The nervous system evolved to enable context-contingent movement.
- The simplest and most efficient biological mechanism for motility, starting with primitive flagella, is an opponent process involving the alternation of agonist and antagonist contractile elements. Since motility was the raison d’être for the evolution of the nervous system, the subsequent evolution of neural processes regulating behavior also employed opponent operations and reflects a balance between facilitation and inhibition. The signs and symptoms of brain injury may therefore either reflect a loss of function or manifest the disinhibition of behaviors that are normally inhibited in a healthy brain.
- Since we are the product of evolution, our central processing unit—our brain—must be Version 1.n. In adapting to a changing envirnonment or to a new niche, organisms need to modify the circuits t bequeathed to them by evolution. They cannot start over with a whole new operating system. As organisms evolve, the neural circuits that subserve reflexes become the building blocks of more complex behaviors. The nervous system routinely goes about its business through an orchestration of these circuits by cortical processes that activate or inhibit them (Easton 1973) Saban and Gabay, this issue).
- Our biological computer, our brain, transmits information much more slowly than a digital computer (at about one-third the speed of sound). To survive, then, our actions must be based on *predictions*, which are based on old news, of the anticipated consequences of our actions. This is achieved through a physiological mechanism of recurrent feedback at all levels of sensory and motor processing. This feedback architecture provides feedback at every processing level on prediction errors that are used to modify the ongoing representation of the current state of the organism. In his classic paper “The reflex arc concept in psychology”, Dewey (1896) referred to this architecture as a form of “continual reconstitution”.
- To facilitate rapid responses, these predictions prime multiple-potential responses that are prepared in parallel and compete with one another.
- Errors made by neurological patients often reflect a failure to resolve this competition to select, or to correctly sequence, a required context-contingent response.
- The dysfunctional behavior of patients with focal brain injury is a manifestation of the post-injury functioning of the neural circuitry in which the injured tissue is embedded, and its contributions to those networks are manifestations of the type of neural computations that the damaged region normally performs, the brain structures it receives inputs from, and the brain structures that it projects to. A patient’s behavior after an acute, sudden injury reflects not just the loss of function of the damaged tissue but also the resulting dysfunction of brain regions that the damaged area is connected with—a phenomenon called diaschisis. As the faciliatory and inhibitory imbalances of the lesioned network re-equilibrate, changes in the function of the circuitry may be compensatory to aid the patient’s functional recovery; otherwise, the new state of the network may contribute to the patient’s problems, making things worse.

## 4. The Effects of Bilateral Damage to the Dorsal Visual Stream: Bálint’s Syndrome

We have seen that visual information that is processed by the primary visual cortex is transmitted in two streams: a ventral pathway distributed across the ventral surfaces of the occipital and temporal lobes and a dorsal stream transmitted chiefly to the parietal lobes. Since the cortices of these two pathways are irrigated by different arteries, a stroke can damage the cortical targets of one of the streams while sparing the other. Consequently, dissociations of the perceptual functions subserved by these two separate streams have been well documented for over a century in patients who sustained strokes in one or the other of these vascular territories (Turnbull 1999; Rossetti et al. 2017).

Damage to the ventral visual stream (especially when bilateral) causes various forms of visual agnosia—impairments in recognizing objects, words, faces, places, and colors—but spares the ability to detect objects, even though the patient cannot recognize them, and the ability to look at them and reach toward them accurately. By contrast, patients with damage to the dorsal stream can recognize an object they are looking at, but they do not know where the object is, cannot direct actions toward it, and do not see a scene containing any other objects at other locations in the scene.

**Figure 1:**
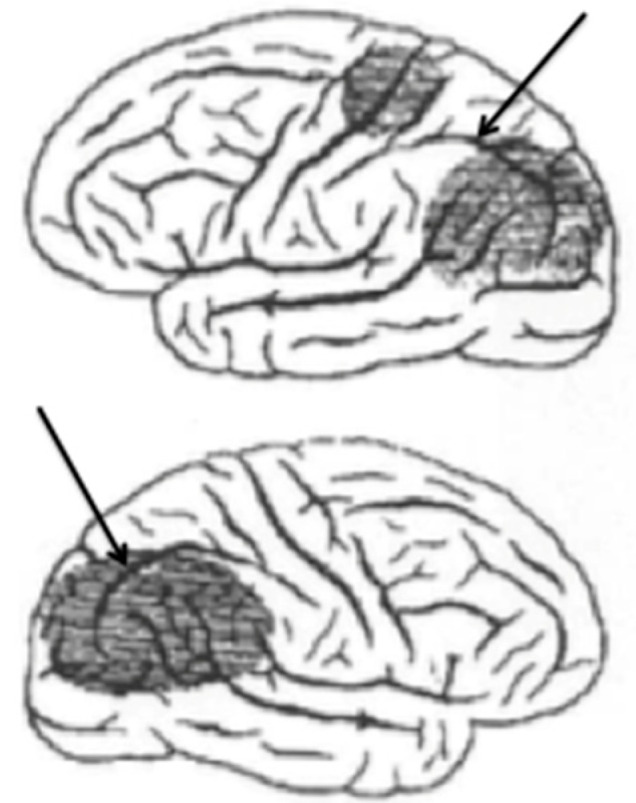
Drawings of the lesions of the patient described by Bálint (1909). Adapted from Rafal (2001, Fig. 3, p. 124). Arrows show the intraparietal sulcus.

Figure 1 shows an adaptation of a drawing published by Rezso Bálint (1909) showing the regions of both parietal lobes damaged in a patient manifesting a syndrome bearing his name (Husain and Stein 1988). These strokes had damaged the dorsal visual stream bilaterally. Arrows have been added to show the intraparietal sulcus that divides the parietal lobe into the inferior and superior lobules.

The intraparietal cortex, which surrounds the intraparietal sulcus, receives and maintains a retinotopic map of visual information transmitted from the primary visual cortex (Silver et al. 2005). As we shall see, in the intraparietal cortex, these visual signals are integrated with copies of motor commands to move the eyes and upper limbs. Damage to intraparietal cortex causes a bilateral disintegration of these two sources of information and engenders a catastrophic derangement of perception and visually guided behavior that manifests as Bálint’s syndrome.

While visual acuity is preserved and patients can recognize single objects placed directly in front of them, they are unable to make sense of or interact with their visual environment. The size of the object does not matter. Patients can see an ant or an elephant, but they cannot see any other objects in the scene. Fleeting objects that they can recognize, but cannot localize or grasp, appear and disappear, and their features jumble together: “… lost in space and, stuck in a perceptual present containing only one object which he or she cannot localize or grasp, a patient with Bálint’s syndrome is helpless in a visually chaotic world” (Rafal 2001, p. 139).

Coslett and Saffran (1991) capture the chaotic visual experience of a patient with this disorder:

“On one occasion, for example, she attempted to find her way to her bedroom by using a large lamp as a landmark; while walking towards the lamp, she fell over her dining room table. Although she enjoyed listening to the radio, television programs bewildered her because she could only ‘see’ one person or object at a time and, therefore, could not determine who was speaking or being spoken to; she reported watching a movie in which, after hearing a heated argument, she noted to her surprise and consternation that the character she had been watching was suddenly sent reeling across the room, apparently as a consequence of a punch thrown by a character she had never seen. Although she was able to read single words effortlessly, she stopped reading because the ‘competing words’ confused her. She was unable to write as she claimed to be able to see only a single letter; thus, when creating a letter, she saw only the tip of the pencil and the letter under construction and ‘lost’ the previously constructed letters”.

(Coslett and Saffran 1991, p. 1525)

This tutorial attempts to make sense of the plight of these patients by considering the origin and functions of the dorsal visual stream. We start by reviewing the two cardinal features of the syndrome: spatial disorientation and simultaneous agnosia.

**Figure 2:**
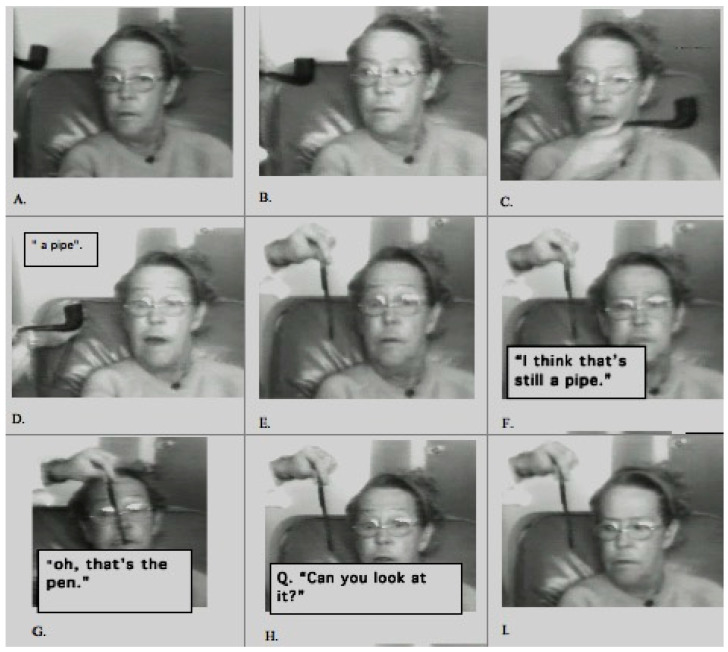
Things appear and disappear. (**A**) This woman, who had recently experienced bilateral parietal strokes, initially failed to notice a pipe that I held to the right of her fixation. (**B**) When I attracted her attention by moving it, she looked it, and she was able to follow it when it was moved to her left (**C**). When the pipe was moved again (**D**), she did not follow it with her eyes and reported seeing the pipe; however (**E**), when the pipe was removed and replaced by a pen held eccentric to her fixation, she failed to look at it, and (**F**) when asked to identify it, reported that “it’s still a pipe”. (**G**) When it was moved into her fixation in the next frame, she correctly identified it as a pen, but when it was then moved again, it disappeared (**H**,**I**) and she could not find it. Adapted from Rafal (2001, Fig. 9, p. 132).

Figure 2 shows a series of sequential images from a video [Video S1, “Balint eye movement for Figure 2”, provided in the Supplementary Materials] of a patient who had experienced recent bilateral parietal strokes and had begun to make a partial recovery. She could recognize an object held directly in front of her, but if it was moved, it would immediately disappear. She was spatially disoriented: when she was asked to rise from her chair and walk across the room, she could not find her way back to her chair.

**Figure 3:**
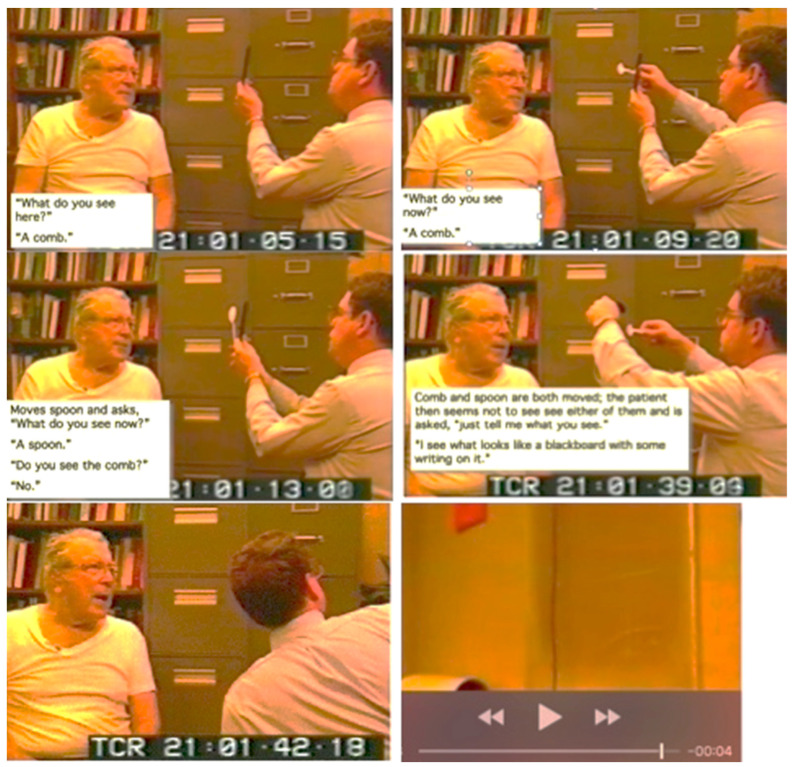
Simultaneous agnosia in a patient with Bálint’s syndrome, showing attentional capture by a distant stimulus. From Rafal (2001, Fig. 5, p. 126). From a video by Perpetua Productions. This patient could only see one object at a time. He could see a comb or a spoon, but not both, even though they were projected on the same region of his retinae. At one point both the comb and the spoon disappeared when his eyes locked on some fine chalk marks on a blackboard behind the examiner.

Figure 3 from this video (Video S2, “Balint Chalk for Figure 3”, provided in the Supplementary Materials) shows a series of images of another patient with bilateral parietal damage. It demonstrates the symptoms of simultaneous agnosia. His visual world contains only a single object. He cannot see a second object placed in front of or behind the first, even though it is projected onto the same region of the retinae. So, he reported either seeing a comb or a spoon, but he never saw both. At one point, his eyes focused on some chalk marks on a blackboard behind the examiner, and both objects he had just seen individually (the comb and the spoon) disappeared. Note, however, that he did not report seeing an isolated white line; he reported seeing a “blackboard with some writing on it”.

The patient shown in Figure 2 was being shown a comb and a pipe, one behind the other, by a doctor wearing a white coat. She reported seeing either the comb or the pipe, but not both. At one point, however, she reported seeing “a white coat” that the doctor was wearing. She then paused for several seconds and said, “I do not know why, I think there’s like a comb and a jacket that kind of thing”. When seeing a comb against the background of the doctor’s jacket, she could not make sense of it, and she reported seeing “a comb and a jacket, that kind of thing”, as if her perception was unable to accommodate seeing more than one object.

Coslett and Lie (2008) showed that not only did a patient with Bálint’s syndrome report seeing only one object in a scene but that the patient was also only able to report one feature of the object, i.e., either its shape or color but not both. For example, he was able to read a word but was not able to correctly report the color of the ink that the word was written in. When the patient shown in Figure 2 was shown an opaque yellow plastic water pitcher (the kind you find at the patient’s bedside in every hospital), she said that it looked like a “pitcher of lemonade”. She correctly reported both the identity of the object (by its shape) and its color but did not correctly integrate the features; therefore, she inferred that the pitcher was transparent and contained a yellow liquid.

In patients with Bálint’s syndrome, the locations of the features of objects jumble together, resulting in illusory conjunctions of features (Friedman-Hill et al. 1995; McCrea et al. 2006). So, for example, when shown two colored objects (letters on a computer screen), a patient with Bálint’s syndrome only reported one letter but often identified it as having the color of the letter that was not reported.

**Figure 4:**
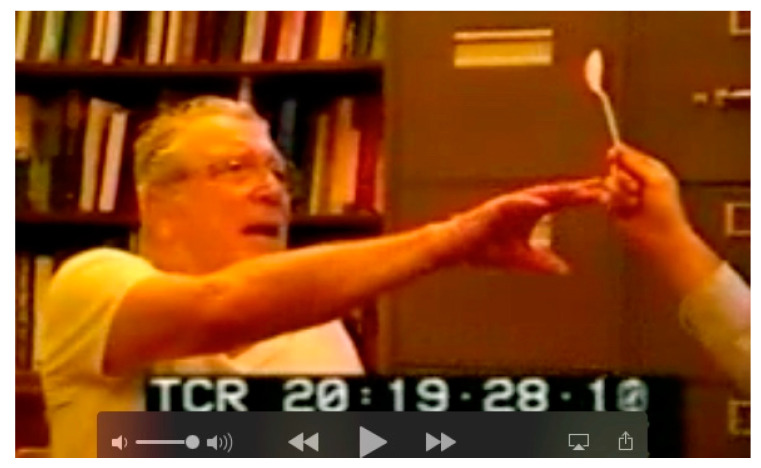
Optic ataxia (misreaching) in a patient with Bálint’s syndrome. Although this patient said that he saw a spoon and was looking at it straight in front of him, he was unable to accurately reach toward it or to orient his hand appropriately to grasp it. From a video by Perpetua Productions.

Neither of the two patients shown in Figure 2 and Figure 3 are optically blind. They are both able to recognize single objects (even very small objects). So, the functions of the ventral visual stream are preserved. However, neither sees a visual scene that contains objects that have spatial relationships with one another and with the observer. There is no there out there, so they are lost in space. The patients can be aware of only one object at a time; they cannot report the location (left, right, up, or down) of objects that they do see, and they cannot accurately reach toward these objects (Figure 4), a symptom that Bálint called optic ataxia (Pisella et al. 2017).

From these clinical observations, we can infer that the job of the dorsal visual stream is to reconstruct a visual scene: a representation of the visual world in egocentric co-ordinates that can be used to control action.

## 5. The Neural Origins of the Dorsal Visual Stream

Unlike the colliculi, each of which receives retinotectal visual signals chiefly from the temporal hemifield of the contralateral eye, the geniculostriate pathway from the lateral geniculate nucleus of the thalamus provides the primary visual cortex of each hemisphere with retinal signals from both eyes (Sylvester et al. 2007). Two channels of visual information are transmitted to the primary visual cortex via the lateral geniculate nucleus of the thalamus. One channel derives from midget retinal ganglion cells, located chiefly in the central fovea of the eye, which have small receptive fields. This information channel transmits high-acuity information from retinal cone photoreceptors to the small-cell parvocellular layers of the lateral geniculate nucleus of the thalamus. The other channel is derived from parasol retinal ganglion cells that have large receptive fields. This information channel transmits low-resolution visual signals from rod retinal photoreceptors that are distributed across entire retina to the large-cell magnocellular layers of the lateral geniculate nucleus.

These two channels both project to different parts of layer 4 of the primary visual cortex and convey different types of visual information. Magnocellular geniculate neurons respond best to low-frequency, low-contrast (i.e., fuzzy) stimuli. They do not exhibit color opponency, but they are highly sensitive to motion. Magnocellular neurons generate transient, phasic responses (i.e., they respond briskly to the onset and offset of visual signals) to sudden changes in the visual environment and thus function as salience detectors. In contrast, parvocellular neurons have small receptive fields affording high acuity, exhibit color opponency, and generate tonic responses to visual stimuli (i.e., they continue to respond as long as the visual stimulus is in their receptive field.)

These two channels remain largely segregated as they project through two different streams of the extrastriate visual cortex. The ventral stream transmits detailed shape and color information from the parvocellular channel that is optimally configured for the discrimination of objects at fixation. It projects to the ventral cortices of the occipital and temporal lobes. The dorsal stream transmits signals from the magnocellular channel that are optimized for detecting luminance flux and motion, especially from the visual periphery. Visual information transmitted in the dorsal stream is useful for triggering an orienting response (to summon eye movements that foveate salient visual signals of potential importance for detailed analysis in the ventral stream), as well as for guiding manual reaching and grasping responses. The dorsal stream projects to the lateral cortices of the dorsal temporal and the parietal and frontal lobes.

**Figure 5:**
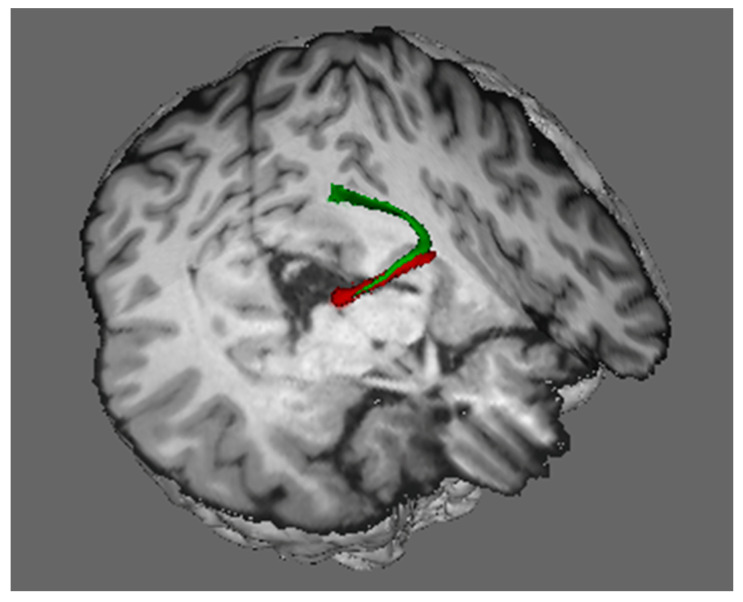
A virtual dissection, using probabilistic diffusion imaging tractography, showing connections between the lateral geniculate nucleus and the superior colliculus (red) and projections from the primary visual cortex to the superior colliculus via the brachium of the superior colliculus (green).

The dorsal visual stream transmits visual signals from the superior colliculus. Intercalated between the magnocellular and parvocellular layers of the lateral geniculate are layers consisting of heterogeneous populations of very small cells called koniocellular cells [meaning “dust” cells (Kaas et al. 1972), named because of their very small size], which are similar in size to other thalamic neurons that project to the cerebral cortex. Although much less conspicuous than the magnocellular and parvocellular layers of the lateral geniculate nucleus due to their small size, the koniocellular layers contain as many neurons as the magno- and parvocellular layers (Hendry and Reid 2000). They receive input from a third class of retinal ganglion (bistratified cells) but also receive afferents from the superficial layers of the superior colliculus (Harting et al. 1978) via the brachium of the superior colliculus (Figure 5). Collicular signals are relayed to the primary visual cortex via the koniocellular neurons in the lateral geniculate synapse in layers 1 and 2. Thus, the projections from the primary visual cortex to the dorsal stream transmit information derived from both the magnocellular layers of the lateral geniculate nucleus of the thalamus and the superior colliculus.

There are also direct projections from koniocellular neurons in the lateral geniculate nucleus to the dorsal stream, specifically to area 5/MT in the posterior medial temporal lobe, which has neurons that respond selectively to the direction of moving stimuli. This projection frequently enables hemianopic patients, who have been rendered blind in one visual field due to the destruction of the primary visual cortex in one hemisphere, to report the presence and direction of fast-moving objects in their blind field—known as the Riddoch effect—though they are unable to ”see” the object that is moving (Zeki and Ffytche 1998).

## 6. The Dorsal Stream Is Embedded in a Neural Circuit with the Superior Colliculus as Its Hub

Having observed the effects of damage to the dorsal visual stream on scene perception in patients with Bálint’s syndrome, we can begin to consider how it does its job. Since our view of the visual scene changes with every movement of the eyes, the experience of a stable and meaningfully coherent environment requires that visual information transmitted from the retina be integrated with continuously updated information about eye position in the orbit. These two types of information, visual signals and eye position signals, are integrated in the intraparietal cortex—the brain region damaged in patients with Bálint’s syndrome.

**Figure 6:**
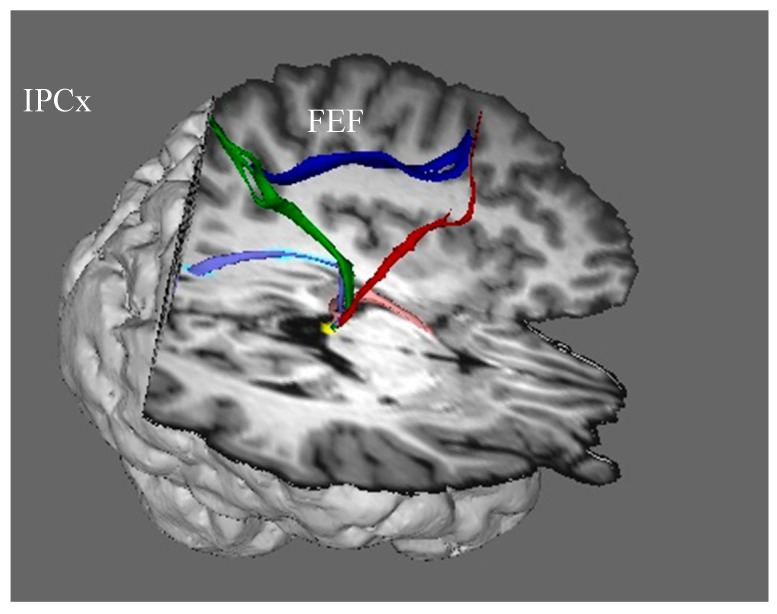
Virtual dissection, with probabalistic diffusion tractography, showing that the dorsal stream is embedded in a network with the superior colliculus, on the back of the midbrain (yellow), as its hub. The dorsal stream refers to projections (not shown) from the primary visual cortex (V1) to the dorsal extrastriate cortex including the intraparietal cortex (IPCx) and the frontal eye field (FEF). The colliculus receives visual signals from the retina both directly, via the retinotectal tract (pink), and from the primary visual cortex (light blue), with both visual pathways entering the colliculus via the brachium of the superior colliculus. In addition to receiving visual signals from the magnocellular and parvocellular layers of the lateral geniculate nucleus, the visual cortex also receives visual signals from the superficial layers of the superior colliculus via the koniocellular layers of the lateral geniculate nucleus (not shown). The intraparietal cortex also receives information about the position of the eyes in the orbit: When the superior colliculus sends eye movement commands to eye movement generators in the brain stem, a copy of the command is sent, via recurrent axon collaterals, to the intraparietal cortex via the frontal eye fields (red). The region of the frontal eye fields that receives eye movement signals from the colliculus projects to the intraparietal cortex as a component of the superior longitudinal fasciculus (dark blue); and the intraparietal cortex, in turn, is connected to the superior colliculus (green). The frontal eye field also sends endogenously generated commands for voluntary eye movements to the superior colliculus—which is the final common pathway for initiating orienting responses.

We have seen that visual information from the retina, which is then projected through the dorsal visual stream, is derived both from the superior colliculus and from the lateral geniculate nucleus. In addition to contributing visual signals to the dorsal visual stream, the superior colliculus also sends copies of motor commands that move the eyes to the intraparietal cortex via the thalamus and the frontal eye fields. These oculomotor signals provide neurons in the intraparietal cortex with continuously updated information about where the eyes are looking (Figure 6).

### 6.1. The Function of the Superior Colliculus Is to Select Behaviorally Salient Targets for Action

In considering how the dorsal stream does its job, we begin with two bumps on the roof of the midbrain (the optic tectum) which, in mammals, are called the superior colliculi. Each colliculus receives direct projections from the opposite eye. A frog sitting on its lily pad has an eye on each side of its head, each of which sees half of the visual field. A frog’s retina is more complicated than ours. It has four types of retinal ganglion cells that convey four channels of information that make it a highly efficient “bug detector” that can detect curved objects that are moving (Lettvin et al. 1959). These signals are transmitted to the contralateral optic tectum that can then implement a context-contingent motor response: orienting toward the object if it is small (prey) or away from the object if it is big (a predator).

In addition to receiving visual input from the visual cortex, the intraparietal cortex also receives information from the superior colliculus about the position of the eyes in the orbits: when the superior colliculus sends eye movement commands to eye movement generators in the brain stem, a copy of the command is sent via recurrent axon collaterals to the intraparietal cortex via the frontal eye fields (the red streamline). The region of the frontal eye fields that receives eye movement signals from the colliculus projects information to the intraparietal cortex as a component of the superior longitudinal fasciculus (dark blue streamline), and the intraparietal cortex, in turn, projects it back to the superior colliculus (green streamline). The frontal eye fields also send commands for endogenously generated voluntary eye movements, directly and via the basal ganglia, to the superior colliculus, which is the final common pathway for initiating orienting responses.

**Figure 7:**
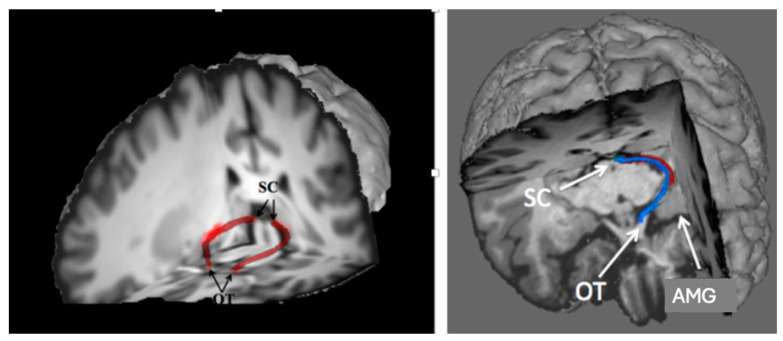
Three-dimensional reconstructions of the retinotectal tract and projections from the superior colliculus to the amygdala. (**Left**): Viewed from the left front, the retinotectal tract (red) in the human brain was virtually dissected using probabilistic diffusion imaging tractography. A seed mask was placed in the optic tract (OT) and a target mask was placed in the superior colliculus (SC). (**Right**): The retinotectal tract in blue, viewed from above, and projections (red) from the superior colliculus to the amygdala (Amg).

In mammals, direct visual input to the superior colliculus is conserved via the retinotectal tract (Figure 7). While the colliculus primarily detects salience signaled by visual transients—like luminance flux or quick or looming movement—in humans, the colliculus can also detect simple shape configurations signaling salience.

For example, in newborn human infants, synaptogenesis has not yet occurred in the primary visual cortex, so the newborn baby is, visually, a collicular creature. But its colliculus is ready at birth to discriminate simple features that ensure that the baby can begin to bond with its parent. Newborns will track a basic face-like stimulus consisting of an oval containing dark circles forming an inverted triangle (Johnson et al. 1991, Figure 8).

Mammals have also retained a subcortical pathway via collothalamic projections from the superior colliculus to the amygdala (Figure 7, right) that bypasses the primary visual cortex. This subcortical pathway mediates orientation toward threatening visual stimuli (Rafal et al. 2015; Koller et al. 2018).

Recall that in prey animals, like the frog or rabbit, the eyes are placed on the sides of the head such that the animal has a wide, panoramic field of vision that is optimized for detecting threats, and each optic tectum/colliculus receives all its visual input from the opposite visual field. In contrast, in the evolution of predatory vertebrates, the eyes migrated to the front of the head such that each eye receives visual signals from both visual fields to enable precise depth perception. In mammals with forward-facing eyes, including humans and other primates, each eye receives information from both the left and right visual fields, and the visual cortex in each hemisphere of the brain (left and right) receives visual information from each eye from the contralateral visual field. However, the retinotectal tract still dominantly projects visual signals only from the contralateral visual field. That is, each colliculus receives most of its retinal input only from the temporal hemifield (that is, from the nasal hemiretina) of the contralateral eye (Sylvester et al. 2007).

Because of these anatomical differences between the retinotectal pathway and the geniculostriate pathway to the visual cortex, temporal/nasal performance asymmetries, when tested under monocular viewing (i.e., with one eye patched), can serve as behavioral markers to assess the relative contributions of the phylogenetically older retinotectal pathway and the phylogenetically more recent geniculostriate pathway to the visual cortex.

For example, when a person (or a monkey) is trained to look at visual targets appearing at different locations in the visual field, two neural mechanisms involving different neural circuitry are activated simultaneously. The retinotectal pathway activates the eye movement system reflexively, but since the participant in the experiment has been instructed to look at the target, there is also a voluntary contribution to performance. In an experiment like this, the results reveal a range of eye movement latencies (the reaction time between the appearance of the visual target and the initiation of eye movement), ranging from very short latency saccades to longer latency saccades. While the longer latency saccades reflect the voluntary, cortically mediated component, the shorter latency saccades are those that were triggered reflexively by direct retinotectal input. In fact, while searching for something else, people frequently make reflexive eye movements toward extraneous visual signals that they are not aware of having seen (Theeuwes et al. 1998). Koller and Rafal (2019) showed that more short-latency, reflexive saccades are elicited by stimuli presented in the temporal hemifield than by stimuli presented in the nasal hemifield. The relative reduction of saccade latency observed when targets are presented in the temporal hemifield reflects an additional reflexive tug on the eyes that pulls the eyes toward the target via the collicularly mediated visual grasp reflex.

Under monocular viewing (with one eye patched), a newborn baby will only reliably orient toward a face-like figure in its temporal hemifield (i.e., with the right eye patched, a baby will orient toward a face in the left visual field but not the right visual field, and vice versa if the left eye is patched) (Simion et al. 1998). Adults continue to show this temporal hemifield bias for orienting toward face-like stimuli (Tomalski et al. 2009). Dodds et al. (2002) demonstrated that a hemianopic patient who was blind in her left visual field after a right occipital stroke was able to localize, with a consistency better than by chance, visual targets that she could not “see” in her blind hemifield when it was presented in the temporal but not the nasal hemifield.

### 6.2. The Superior Colliculus Maintains a Salience Map That Is Used to Select a Target for Action

The salience map that is generated by the superior colliculus is a physical map of neurons across the surfaces of each of the two bumps. A neural salience map is a priority map in which the degree of neuronal activation at a given location on the map encodes the degree to which that location is prioritized for action. Some events, like abrupt onsets, moving or looming visual stimuli, evoke a brisk activation of collicular neurons and are thereby implicitly encoded as being salient. When one location on the neural salience map reaches a threshold, a command is sent from neurons in the deep layers of the colliculus to brainstem eye-movement generators to execute an orienting movement of the eyes and/or body; these neurons inhibit other eye-movement neurons with which they are competing.

Each location on the neural salience map of the superficial layers of the colliculus corresponds to a topographic, or retinotopic, map in the retina. Thus, the colliculus salience map provides a place code that encodes the location of salient visual stimuli in eye-centered coordinates. Neurons at each point on the colliculus receive visual signals via the retinotectal tract (and, in mammals, from the primary visual cortex) from an organized map of the visual environment in retinal coordinates. Visual input to the fovea (straight-ahead vision) is projected to the rostral (top) of the bump, while retinal input from progressively more peripheral locations in the visual field project to progressively more caudally located neurons on the bump.

The colliculus neurons that receive visual signals from the retina via the retinotectal tract (and from primary visual cortex) are located in the superficial layers of the superior colliculus (i.e., near the surface of the bump). Deeper than these visual neurons are neurons that also respond to visual stimuli, as do the neurons above them in the superficial layers, but in addition, when these deeper neurons are activated, they send commands to brainstem eye-movement generators to execute conjugate eye movements (i.e., movements made by both eyes) toward the selected salient location. These neurons not only send axons to the brainstem to activate eye movements but also send recurrent, collateral axons back to the visual system to provide updated information about the position of the eyes in the orbits. This information is integrated with visual information in the dorsal stream to compute the locations of salient objects in the environment in egocentric coordinates that can be used to guide actions.

### 6.3. The Superior Colliculus Is the Hub of the Dorsal Visual Stream

The superior colliculus is not only the phylogenetic origin of the dorsal visual stream; it remains its hub in mammals. It deserves this designation for the following reasons:

- In order to encode the location of an object in a neural representation that can be used to control action in egocentric coordinates, the intraparietal cortex of the dorsal stream must integrate two types of information: retinotopic visual information and information about the position of the eyes in the orbits. The superior colliculus provides both types of information.
- The salience map computed by the colliculus weights both visual salience based on external stimulation and the demands of voluntary, goal-directed actions. For example if, while driving, I am approaching an intersection at which I plan to turn right and as I am about to look to the right, I detect a sudden movement on the left out of the corner of my eye (a car running a red light, i.e., a large, looming threat), that stimulus will win the competition for salience and I will look to my left.
- It is the final common pathway for implementing orienting responses.
- The colliculus not only resolves the competition for behavioral salience and executes a response to look at the location that is selected as the most salient; it also transmits this information to the cortex of the dorsal visual stream.
- This information is critical for the dorsal stream to do its job for two reasons. First, it provides the cortex of the dorsal stream with continuously updated information about eye position in the orbit. It also tags the location toward which the eye movement has been selected as being salient, and biases that location for further processing using a signal that it transmits back to the salience map in the colliculus (the green streamline in Figure 6).

Saban and Gabay (2023) have highlighted the historical “cortico-centric” bias of neuroscience and neurology in which the cerebral cortex is construed as the top of the cognitive hierarchy while subcortical structures are relegated to bottom of the hierarchy, their function construed as only the implementation of the product of the cognitive operations of the cortex. Saban and Gabay challenge this historical bias and contrast three models of cortico-subcortical processing: a *solitary cortex* model, a *cortical superiority model, and a dynamic network model*. By cortico-centric accounts, for example, in the course of evolution, the visual salience map “migrated” from the superior colliculus to the visual cortex (Li 2016).

Song et al. (2011) explicitly challenged the conventional cortico-centric, hierarchical model of orienting behavior. On this account, target selection occurs at the level of the cortex and is effector-independent. That is, the cortex selects a target to prioritize for responding, while the specific response (an eye movement or a reach toward the target) is implemented by lower structures in the hierarchy, each of which implements the response of a specific effector. So, for example, the cortico-centric hierarchical model posits that the superior colliculus (which is involved in generating eye movements but not in executing reaching movements) is involved in selecting targets for eye movements but not in targeting reaching responses.

Song et al. (2011) trained a monkey to reach (with the eyes held steady at fixation) toward the first of two targets to appear, one on the right and one on the left, and in some trials, the two targets onset simultaneously. In these simultaneous trials, the monkey’s responses were random (50%/50%). When one colliculus was transiently inactivated with muscimol (a GABA agonist), however, the monkey was strongly biased to reach toward the target presented in the visual field ipsilateral to the inactivated colliculus. A second experiment showed that this did not happen because collicular inactivation slows or impairs processing of the visual stimulus. In a second monkey who was trained to reach toward the second target to appear, the same performance was observed. The monkey was still less likely to correctly reach toward the second target if it appeared in the visual field contralateral to the inactivated colliculus.

These observations demonstrate that collicular inactivation does not impair the perceptual processing of visual stimuli. That is, a visual stimulus contralateral to the inactivated colliculus was not processed more slowly or detected later than a visual stimulus in the unaffected visual field. Rather, collicular inactivation renders contralateral visual stimuli less salient and of a lower priority for engendering a response. Moreover, the same response bias against orienting toward the visual field contralateral to the inactivated colliculus was also observed when the monkey was tested with central arrow cues rather than peripherally appearing visual targets. Thus, the superior colliculus is not only the final common pathway for selecting visual targets for eye movements (whether initiated reflexively or voluntarily); it is also responsible for selecting targets for reaching movements as well.

It should also be noted that the role of the superior colliculus in controlling orienting behavior is not restricted to visual targets. The deep layers of the superior colliculus also receive auditory and tactile input, and the colliculus is the final common pathway for orienting toward salient tactile and auditory stimuli as well as toward visual targets. Moreover, it should be emphasized that collicular control of orienting is not restricted to eye movement. Motor commands from the colliculus are also transmitted via the tectospinal tract to initiate orienting movements of the head and trunk.

### 6.4. Visually Guided Behavior in Progressive Supranuclear Palsy

**Figure 8:**
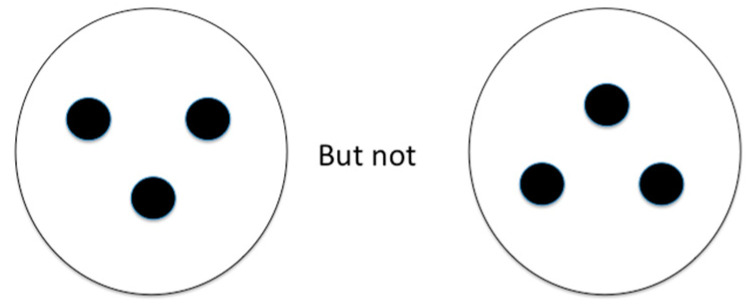
Newborn infants will track the moving face-like figure on the left but not the inverted figure on the right. Also, a baby will not track the figure on the left if the contrast is inverted (white dots forming a triangle on a black background).

People who have sustained damage to the primary visual cortex in both hemispheres are rendered cortically blind. They are not consciously aware of any visual stimuli. So, it is perhaps understandable that neurology textbooks assign no role for the superior colliculus in visual perception, relegating the colliculus to some role in executing eye movement but being otherwise vestigial, with its role in seeing having migrated to the primary visual cortex. We’ve seen that the colliculus provides some primitive perceptual abilities that, in newborns, bootstrap visual development (Figure 8) and that there is a pathway that transmits visual threat signals directly to the amygdala, bypassing the primary visual cortex (Figure 7). Some patients with cortical blindness manifest “blindsight”, affording coarse location information about visual signals they are not consciously aware of (Dodds et al. 2002). But does the superior colliculus make a contribution to scene perception in adults?

The vital role of the superior colliculus in manifesting visually guided behavior in humans is strikingly evident in neurological patients with degeneration of the superior colliculi (Rafal and Grimm 1981). Progressive supranuclear palsy (PSP) is a tragic, progressive, and ultimately fatal condition involving the degeneration of multiple subcortical nuclei and with many of the clinical features of Parkinson’s disease. One of the pathological features that distinguishes it from Parkinson’s disease is degeneration of the dorsal midbrain, including the superior colliculi. Although visual acuity per se is preserved, patients behave as if they were blind. The presenting complaint is often poor eyesight, and patients have often seen eye doctors—and have a drawer full of glasses that have done them no good—before any obvious neurological symptoms are evident.

A clinical feature that distinguishes PSP from Parkinson’s disease is what Campbell Posey described as a “peculiar fixity of gaze” (Posey 1904). This produces a striking countenance with lid retraction and a wide-eyed, fixed, and unblinking gaze, giving a look of “perpetual astonishment” (See Rafal 1992, Fig. 11-1, p. 204). As the disease progresses, the eye movements become progressively paralyzed, but early on, patients just stop using their eyes, and a global derangement of visually guided movements supervenes as the disease progresses.

What friends and family often notice first is that the patient stops looking at them when in conversation. When I first saw a patient with PSP, I was particularly struck with this lack of social orienting because of my experience as an ROTC cadet a decade earlier. The problem was standing at parade during an inspection. The way this works (the Army way) is that while you are standing at attention with your eyes fixed straight ahead, the drill instructor walks up from the side until his face is inches from yours, but a few inches to the side. And he yells at you. What the cadet must not do is look at the drill instructor while being inspected—and yelled at. I can report that his is very hard to do, or rather, to not do. It takes a great deal of discipline to not look at a looming face approaching you from the side. And, of course, from the Army’s point of view, instilling discipline is the point of the exercise.

So, a decade later, I am talking to a patient with PSP about his medication, and although he is answering me, he does not look at me. When I try my drill inspector imitation and lean toward him so that I am inches from his face, he does not look at me. I pulled back and just asked him, “can you look at me”? He did so promptly (See Rafal and Grimm 1981, Fig. 1, p. 1510). So, although his eyes were not paralyzed, automatic reflexive orienting toward a salient stimulus was lost, as were other spontaneous visually guided behaviors.

For example, a patient struggling to open a milk carton on a hospital dinner tray may not, at first, automatically look down at it, only doing so after experiencing frustration at failing to complete the task. Note here that there is no loss of awareness of people or things. The patient who failed to orient toward me while we were conversing knew I was there; he just did not look at me until he was asked to do so. A patient who experiences difficulty when opening a milk carton knows it is there and roughly where his hands can find it. Patients simply do not automatically use their eyes. Normally, a looming visual stimulus, or any movement in the visual periphery—a potential threat—automatically triggers an automatic visual grasp reflex that pulls the eyes toward the threat. Reflexive shifts in attention toward the onset of a visual stimulus are impaired in patients with PSP (Rafal et al. 1988).

## 7. How Does the Dorsal Visual Stream Reconstruct a Visual Scene?

“The eye is not our servant; it is our ambassador”.

Simon Ings (2008)

Try this: close one eye and, with your finger, press on the eyelid of your viewing eye to move it. What you will see is that the visual world jumps. This is what you would expect to see because when you move your eye, all the visual input translates across the retina. So, why does the world not jump when you make eye movements? The reason is that when you make any eye movement, the neurons in the superior colliculus that send motor commands to brain stem eye-movement generators also send a collateral axon to the visual system. This information enables the visual cortex to take the eye movement into account so that what we perceive when we move our eyes to view a scene is a stable world containing objects that have stable spatial relationships with the observer and with each other.

**Figure 9:**
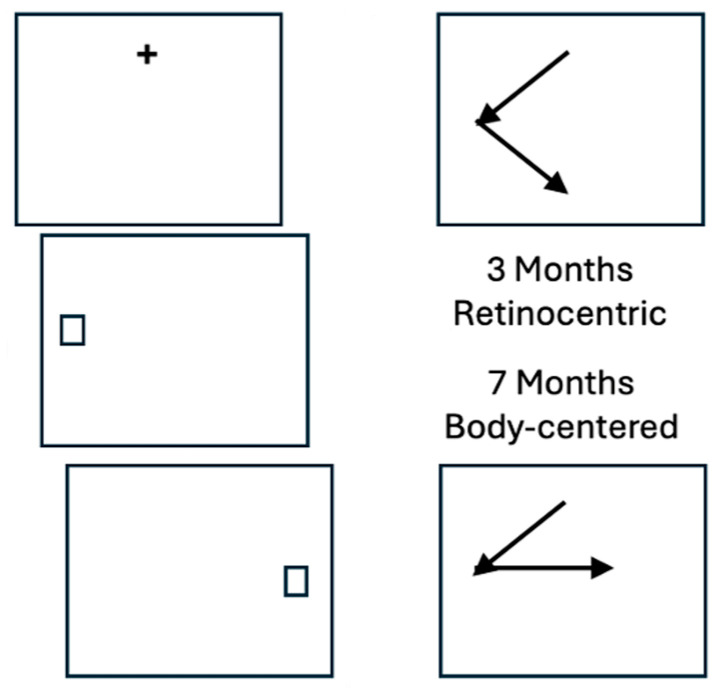
The performance of 3- and 7-month-old infants on a double saccade task (Gilmore and Johnson 1997). (**Left**): The temporal sequence of a trial in the double saccade task, shown from top to bottom. Participants fixate the + at the top of the display. Simultaneous with the offset of the +, two targets are presented in sequence, with the first of the two targets appearing randomly on the left or right. Participants are instructed to look at the locations of the two targets in the order they appear. In this illustration, a target on the left is followed by a target on the right. Critically, both targets are flashed only briefly in quick succession such that both targets disappear before the first eye movement can be initiated. (**Right**): The performance of 3-month-old (top) and 7-month-old (bottom) infants on the double-step saccade task. The 3-month-old infants made eye movements toward the location that the target had appeared on their retinae. The 7-month-old infants’ performance reflects the effect of an extra-retinal signal (the effect of eye movement) such that the second saccade is made to the actual location in the scene where the second target had appeared.

Figure 9 shows the performance of three- and seven-month-old infants (Gilmore and Johnson 1997) on a double-step saccade paradigm (Hallett and Lightstone (1976)). In this variant of the task, observers fixate a + and, when the + sign disappears, are very briefly presented with two visual stimuli (one in the right visual field and one in the left visual field) in rapid succession. The order is varied from trial to trial so the participants cannot anticipate which stimulus will appear first; critically, the presentation is so brief that both stimuli disappear before an eye movement is initiated to the first stimulus. So, in Figure 9, for example, the first stimulus appears in the receptive field of ganglion cells in the left lower quadrant of each retina, and the second appears in the receptive field of ganglion cells in the right lower quadrant of each retina.

The infants looked toward each stimulus in the order in which it appeared. The average three-month-old infant has not yet learned that when it moves its eyes, the visual world does not move. So, when it executes an eye movement to the second stimulus, it looks toward the retinal location where the second stimulus had been before the first eye movement. By seven months of age, a baby has mastered “sensorimotor contingency” (O’Regan and Noë 2001), i.e., the ability to predict the effects of movement on perception. The baby has learned to use the efference copy of motor commands that are transmitted from the colliculus to the visual system to update the location of the second stimulus in body-centered rather than retinal coordinates.

Primates have an advantage over quadrupeds in learning these sensorimotor contingencies because they can easily see their forelimbs. Babies spend a lot of time looking at their limbs. If a baby’s eyes move while the baby is looking at their hand, the image of the hand translates across the retina, but the baby also receives conflicting proprioceptive signals from their limbs that the limb has not moved. Therefore, the baby experiences a conflict between visual and proprioceptive information. This conflicting proprioceptive information facilitates the baby’s ability to utilize efference copies of the eye movement motor commands to reconcile the conflict arising from the translation of the visual world on the retina. Young infants are also aided in mastering this sensorimotor contingency by an infantile reflex called the tonic neck reflex. When an infant’s head is turned toward one side, the arm on the side toward which the baby is looking is extended, putting the arm in view.

**Figure 10:**
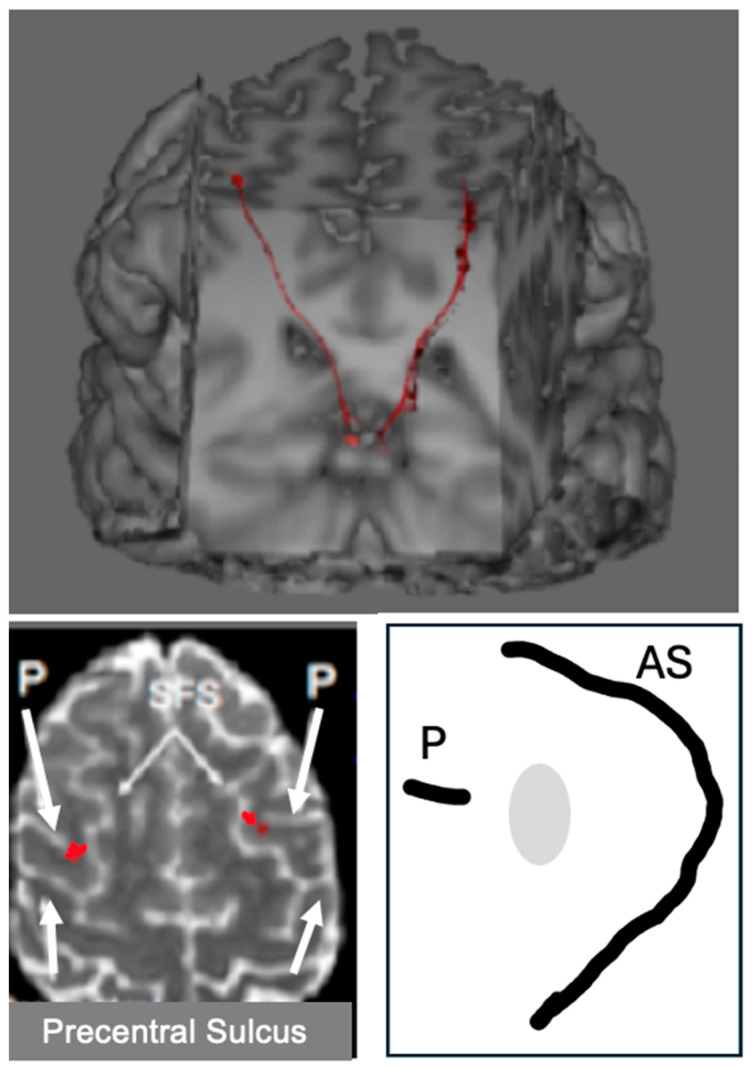
(**Top**) A virtual dissection, using probabilistic diffusion imaging tractography, of connections (red) between the superior colliculus and the frontal eye fields (FEF). The FEF is located on the posterior part of the middle frontal gyrus at the intersection of the superior frontal sulcus (SFS) and the precentral sulcus. (**Bottom**) The figure on the left shows the termination (red), in the FEF, of projections from neurons in the mediodorsal thalamus that transmit copies of eye movement signals from the deep layers of the superior colliculus. The bottom of Figure 10 shows that the termination point (red) of this pathway from the colliculus to the frontal eye field in the human brain (**bottom left**) corresponds closely with that demonstrated electrophysiologically by Sommer and Wurtz (2004) in two monkeys (**bottom right**). When comparing sulcal landmarks in the two species, note that in the monkey brain these projections synapse on FEF neurons located midway between the end of the principal sulcus and the curve of the arcuate sulcus (gray ovals). In the human brain, the homolog of the arcuate sulcus is formed by the intersecting superior frontal and precentral sulci. The sulcus in the human brain, labeled “P” in the bottom left figure, may be a remnant homolog of the monkey principal sulcus (P).

Now, let us take a look under the hood at the neural machinery that implements this ability to see a stable visual world when we move our eyes. Since the superior colliculus is the final arbiter for selecting a saccade target and for generating commands to move the eyes, the collicular neurons generating those commands to the brainstem send axon collaterals upstream to signal the dorsal visual stream that the eyes have moved. Sommer and Wurtz (2008) demonstrated, in monkeys, that these copies of the motor commands are transmitted to the frontal eye field via relay neurons in the lateral border of the mediodorsal nucleus of the thalamus. By inactivating thalamic neurons that receive copies of the eye movement signals from the colliculus, they confirmed that these neural signals from the colliculus are used to update the visual cortex that the eyes have moved. Sommer and Wurtz (2008) tested monkeys on the double-step saccade task before and after the inactivation of the thalamic neurons transmitting signals from the superior colliculus to the cortical frontal eye field. After this inactivation, the monkeys could not execute accurate saccades toward the second target (Sommer and Wurtz 2008). Figure 10 shows the pathway from the superior colliculus to the frontal eye fields in the human brain, and it shows the termination of this pathway in the frontal eye fields of both humans and monkeys.

The frontal eye field then transmits a copy of the eye movement command signals to neurons in the lateral intraparietal cortex. In the lateral intraparietal cortex, the receptive fields of many neurons shift when a saccade is made such that those visually responsive neurons will respond to a visual target that falls outside of their receptive fields but will fall in their receptive fields after the saccade is made. Some lateral intraparietal cortex neurons will manifest this shift even before the saccade is initiated—a phenomenon called predictive or anticipatory remapping (Colby et al. 1995; Merriam et al. 2003). While this shift is sometimes referred to as a remapping of the cell’s receptive field, an alternative account suggests that the efference copy signals act as “attentional pointers” that transfer activity to other locations to update the salience map (Cavanagh et al. 2010). For a review of putative physiological mechanisms for updating attention and eye movements, see Marino and Mazer (2016).

Neurological patients with unilateral damage involving the lateral intraparietal cortex fail, after making an accurate first saccade toward the contralesional field, to make an accurate eye movement to the second target to appear in the double-step saccade task (Duhamel et al. 1992; Heide et al. 2004). And as we have seen, bilateral damage to the intraparietal cortex deprives patients with Bálint’s syndrome of reliable location information about objects in a scene—and so the scene disappears.

The remapping of lateral intraparietal cortex neurons’ receptive fields, based on the information from efference copies of motor commands, thus permits an observer to generate a map of the visual scene in egocentric coordinates that is continuously updated across eye movements.

But just what is it that is “remapped” by the lateral intraparietal cortex neurons? Gottlieb et al. (1998) showed that the entire visual scene is not updated by lateral intraparietal cortex neurons after each eye movement. A lateral intraparietal cortex neuron responds when a stimulus enters its predicted receptive field after an eye movement only if that stimulus is a behaviorally relevant target. If the stimulus that enters the neuron’s field is not a target for the second eye movement, its receptive field is not remapped, and the neuron does not respond. Note here that the responses of an intraparietal neuron reflect which objects in a scene have already been determined to be behaviorally salient. These neurons are the recipients of attentional signals, not the sources of them. The intraparietal cortex is part of a larger attentional network that includes, among other structures, the temporo-parietal junction and the ventral prefrontal cortex (Corbetta and Shulman 2011).

Gottlieb et al. (1998) concluded that “under ordinary circumstances the entire visual world is only weakly represented in lateral intraparietal cortex. The visual representation in lateral intraparietal cortex is sparse, with only the most salient or behaviorally relevant objects being strongly represented”. So, like the superior colliculus, the lateral intraparietal cortex encodes a continuously updated salience map of stimuli currently selected as action targets. Neurodisruption of the lateral intraparietal cortex in human observers via transcranial magnetic stimulation disrupts the remapping of visual salience after an eye movement (van Koningsbruggen et al. 2010), as does damage to the lateral intraparietal cortex in neurological patients (Sapir et al. 2004).

Lateral intraparietal cortex neurons of the dorsal stream enable visual “indexing”: the continuous updating of markers of the current locations of currently behaviorally salient stimuli. This indexing enables observers to track the locations of multiple targets as they move in relation to one another while ignoring irrelevant distractors in the visual environment. These location markers are opaque to the features of the objects that they index. The markers reference only that the objects at the tagged locations have been assigned priority for imminent action, and they contain no additional information about their referents (Pylyshyn 1989). This indexing mechanism maintains a salience map that affords rapid and preferential access to the indexed objects for the further processing of features such as color, texture, and shape.

## 8. Seeing without a Scene: Bálint’s Syndrome Reconsidered

The sensorimotor integration, in the lateral intraparietal cortex, of retinotopic visual information and concurrent information about eye position in the orbit provides scaffolding for the indexed locations of currently behaviorally relevant objects. This scaffolding encodes the spatial relationships of currently behaviorally relevant objects with one another and with the observer. How does this help us understand the plight of patients with Bálint’s syndrome who can see single objects but cannot see scenes?

Consider the kind of closed-circuit TV system that allows police to solve crimes like the Boston Marathon or London subway bombings. Each camera in the closed-circuit network continuously records a visual image of an area with a timestamp. Since the region of space that each camera is aimed at is known, the events in time can be reconstructed: Person A left a knapsack containing a bomb at location 1. By reviewing the images from other cameras in the network, a time series can be reconstructed to determine where Person A came from before the bomb was planted and where Person A went subsequently.

Now, what happens if the crime analyst is deprived of any information about which area is viewed by each of the cameras in the network? All that would be available is a disconnected series of images of different objects/people that have no spatial relationships with one another. This, you can imagine, is the experience of a patient with Bálint’s syndrome, in which random objects flit in and out of view in a visual world with no there out there. Lacking a representation of the spatial relationship between objects and the observer, there are no other locations and hence, no “space” and no other objects.

**Figure 11:**
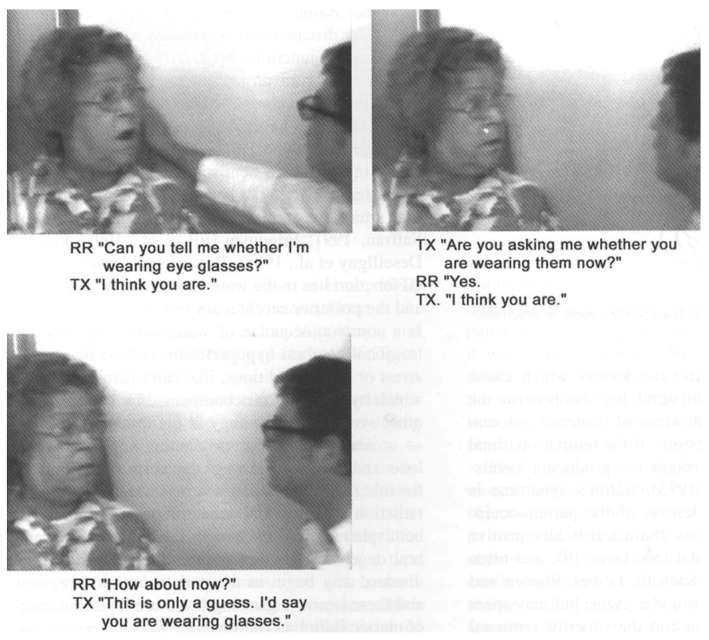
Series from a video of the same patient with Bálint’s syndrome shown in Figure 2. (**Top**): I first asked the patient if I was wearing glasses, and she replied, “I think you are”. (**Middle**): I then ducked away, took off my glasses, and asked her again if I was wearing glasses (**Middle**), and she replied that she thought I was wearing glasses. (**Bottom**): After ducking away again and putting on my glasses, she had “only a guess”. Although my glasses and my face were plotted at the same location on her retinae, she could see my glasses, or she could see my face, but she could not see both (Adapted from Rafal 2001, Fig. 2, p. 123).

Of course, the closed-circuit TV analogy does not fully capture the perceptual experience of patients with Bálint’s syndrome. First, we have a single, mobile cyclopean eye with movements that must be continuously updated, and integrated in time, with retinotopic input; our perception is not experienced as a series of snapshots in which each snapshot denotes the present but rather as a rolling timeline in which prediction and postdiction continuously refresh what we “see” (Hogendoorn 2022). Even when two objects (at different depths) are projected on the fovea, the patient may only see one of them (Figure 11). Recall that Gottlieb et al. (1998) observed that neurons in the lateral intraparietal cortex, which remap the locations of objects in environmental coordinates with each eye movement, only remap the positions of objects that are immediately rendered salient because they are task-relevant; objects at a different distance from the patient than the task-relevant object, although plotted on the same retinal location, are no more privileged to be remapped for sparse indexing in the visual scene than are objects projected at a different location on the retina, nor can patients generate a mental representation of the visual world. For example, the patient reported by Holmes and Horrax (1919) was not able to describe a familiar route from his home. The patient reported by Coslett and Saffran (1991) was able to visualize a route with her eyes closed but not if her eyes were open.

To understand the umwelt of a patient with Bálint’s syndrome, who can see objects but not scenes, we need consider: what is a scene? A useful starting point is to examine the effects of the unilateral disruption of dorsal stream function, a syndrome called hemispatial neglect.

## 9. Unilateral Lesions of the Dorsal Steam: Hemispatial Neglect

**Figure 12:**
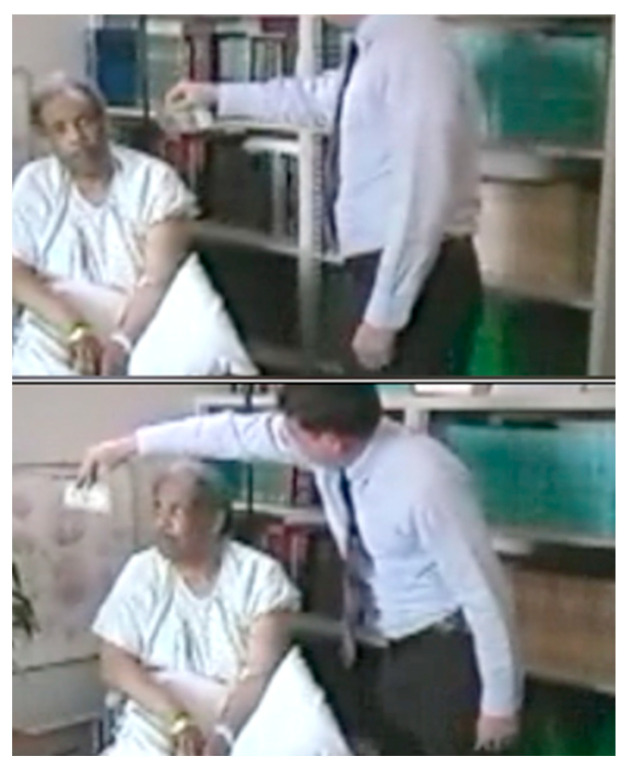
Hemispatial neglect in a patient with hemispatial neglect (See text) (adapted from a video by Perpetua Productions).

This video (Video S3, “Social Neglect Video” from Perpetua Productions, provided in the Supplementary Materials) shows a patient with hemispatial neglect who had experienced a stroke damaging his right parietal lobe ten days earlier. In this video, I have removed my shoes and quietly approach him from his left. He does not orient toward me as I approach. When I call his name, he replies—but he does not look at me. When I then ask him to look at me, he does so, but when I then move slightly further to his left, he does not follow me. Perhaps I am not very interesting? I next try it with a USD $1 bill (Figure 12 and Video S4, “$ Neglect Video” from Perpetua Productions, provided in the Supplementary Materials). He still does not orient toward the money on his left, but when I move it into his right visual field, he orients briskly.

**Figure 13:**
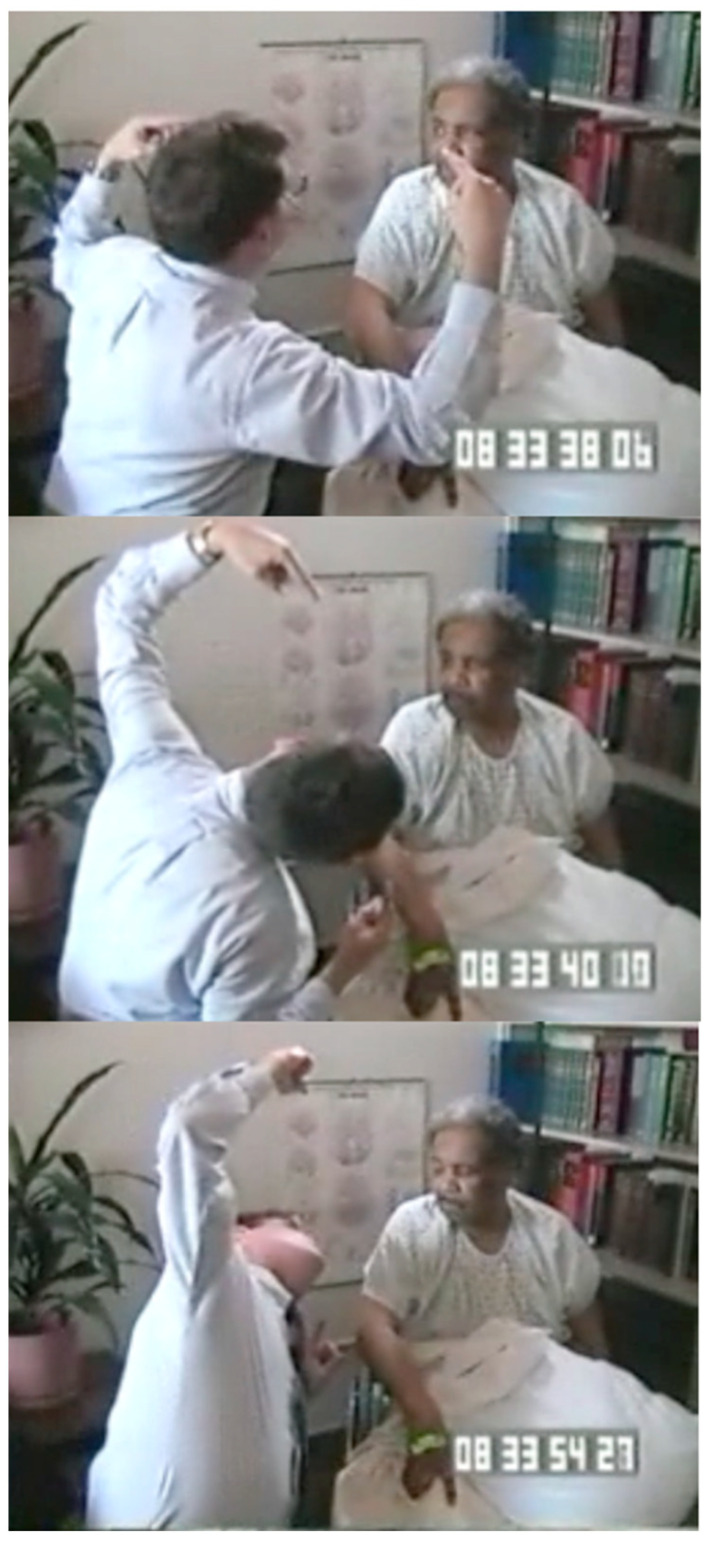
Object-based visual extinction. (**Top**): Both fingers wiggle. The patient looks to the finger wiggling on his right, says “your left hand”, and does not detect the finger wiggling on his left. (**Middle**): When the examiner rotates counterclockwise and wiggles fingers on both hands, the patient looks upward, and he says, “your left hand”. (**Bottom**): When the examiner rotates clockwise and wiggles fingers on both hands, the patient looks down, and he says, “your left hand”. There is neglect of the left side of the object that the patient is viewing (the examiner) regardless of where the left side of the object is in retinotopic coordinates (adapted from a video by Perpetua Productions).

The indexing mechanism described above (Pylyshyn 1989), which maintains scaffolding through which multiple task-relevant objects can be tracked as they move relative to the observer, enables an observer to tag and track multiple objects as they move relative to one another. If the task at hand, however, requires tracking a moving object that changes shape as it moves, the indexing mechanism can tag task-relevant parts of the object’s skeletal structure (Wilder et al. 2011). The object that the patient is attending to in Figure 13 is the examiner, and the task-relevant parts are the examiner’s two hands and face (which the patient is being asked to look at throughout the task).

Figure 13, which is taken from a video (Video S5, “Axis-based extinction” from Perpetua Productions for Figure 13, provided in the Supplementary Materials), demonstrates the operation of this indexing mechanism in a patient with hemispatial neglect who is being tested for visual extinction. His visual fields are being assessed using a bedside test called confrontation testing, which is used to check for visual impairment in different parts of the visual field. In the video, before the sequence of events shown in Figure 13, the examiner raises both of his arms to start the test, and the patient promptly looks to the hand raised in his right (ipsilesional) visual field, demonstrating a bias to orienting rightward. To start the test, the examiner then instructs the patient to “look at me”, and the patient returns his gaze to fixate the examiner’s face. While holding his hands in the patient’s left and right visual fields, the examiner instructs the patient to “tell me when you see a finger wiggle”. The patient was able to detect a finger wiggling in either his right or his left visual field, demonstrating that his retinotopic visual fields were intact. This observation confirms that the primary visual cortex was not damaged in this patient and that he is not blind in the contralesional visual field.

In the sequence shown in Figure 13, the examiner next wiggles both fingers simultaneously. As shown in the top figure, the patient reports “your left hand” and looks to his right, failing to detect the finger wiggling in his left visual field. This clinical sign is called visual extinction because a competing stimulus on his right causes the contralesional stimulus to his left to be extinguished from awareness, even though he had been able to detect it when it was presented without a competing ipsilesional stimulus.

Figure 13 also illustrates axis-based neglect (Driver et al. 1994). In the video (Video S5), the examiner then rotates his body to the right so that both of the examiner’s hands are in the midline (one above and one below, so that neither wiggling finger is in a contralesional location relative to the other wiggling finger). Fingers on both hands are wiggled, with the location of the previously extinguished right hand now below the patient and the previously detected left hand above the patient. The patient looks up and detects the left hand wiggling and again fails to detect the wiggling right hand. Then, when the examiner’s body rotates to the left such that the examiner’s hands in the lower and upper visual fields are reversed, the patient looks down and reports that the examiner’s left hand is wiggling while the examiner’s wiggling right hand is “extinguished” from awareness.

In this demonstration, the indexing mechanism has indexed the examiner’s face and left hand and tracks these indexed features as they rotate. The examiner’s right hand (that was initially in the patient’s left visual field) was not indexed and remains outside the patient’s awareness. Tipper and Behrmann (1996) demonstrated that if a patient with hemispatial neglect views a barbell figure rotated 180 degrees, the patient is then slower to detect a target appearing at the end of the barbell in the right visual field.

The clinical sign of visual extinction demonstrated in Figure 13 is one sign of hemispatial inattention caused by the unilateral disruption of the dorsal visual stream. It reflects the biasing of an opponent process between the two hemispheres of the brain, resulting in both a bias against orienting toward contralesional stimuli and a bias toward orienting toward ipsilesional stimuli. Hyper-orienting toward ipsilesional stimuli has been demonstrated by showing that the neurodisruption of the intraparietal cortex of one hemisphere using transcranial magnetic stimulation not only reduced detection of contralateral stimuli but also enhanced the detection of ipsilateral stimuli (Hilgetag et al. 2001).

Axis-based neglect, demonstrated by the continued extinction of part of an object as its axis rotates, reflects a biased competition that is determined not by the retinotopic visual field but rather by the locations of the competing objects, or object parts, relative to one another in a reference frame determined by the currently task-relevant objects that have been indexed. That is, if two objects are competing for access to be incorporated into a perceived visual scene, the contralesional of the two objects is more likely to be excluded from awareness. Regardless of where two competing objects are located in relation to the patient’s direction of gaze, the leftward (contralesional) of the two objects is disadvantaged and more likely to be extinguished from awareness (Posner et al. 1987). Why should this be so?

### 9.1. Pathophysiology of Hemispatial Neglect

Pisella and Mattingley (2004) have highlighted two separate pathophysiological mechanisms contributing to the clinical phenomenology of hemineglect. One factor results from the simple fact that parietal damage in one hemisphere results in there being more parietal neurons remaining that respond to stimuli in the ipsilesional visual field than those that respond to stimuli in the contralesional visual field. Parietal lobe neurons have large receptive fields. Many respond to visual signals throughout most of the contralesional field visual field and even some of the ipsilesional visual field. So, patients with parietal lobe damage in one hemisphere have fewer neural resources for processing information in the contralesional visual field than in the ipsilesional visual field. This results in a detectability gradient, with patients progressively less likely to detect visual stimuli from the most ipsilesional to the most contralesional locations (Behrmann et al. 1997).

Even after optimal recovery from hemispatial neglect, this laterality bias is never fully compensated. Machado and Rafal (1999) measured line bisection errors in groups of patients with chronic parietal lobe damage. None of these patients had any residual clinical signs of neglect or visual extinction and had not for many years. Nevertheless, the patients with right parietal damage, on average, bisected lines very slightly (on average, 2 mm) to the right of the middle, whereas patients with left parietal damage bisected lines slightly (on average, 2 mm) to the right of the middle.

The second factor is a failure to correctly remap the locations of objects after contralesionally directed eye movements (Pisella and Mattingley 2004). Many perceptual impairments caused by parietal damage may be best understood as resulting from the mislocalization of visual objects in an egocentric representation of space due to defective remapping after each contralesionally directed eye movement. Defective remapping results in a degradation of reliable information about the position of the eye in the orbit—information that is necessary to generate a continuously updated representation of the visual scene.

The syndrome of hemispatial neglect can occur after damage to a variety of brain regions in the temporal, parietal, or frontal lobes (Corbetta and Shulman 2011). Recovery from acute hemispatial neglect, however, has been linked to the functional recovery of the intraparietal cortex (Corbetta et al. 2005), and patients are less likely to recover if the brain damage interrupts white matter connections between the frontal eye field and the intraparietal cortex (Thiebaut-de Schotten et al. 2012). White matter damage that interrupts the transmission of remapping signals from the frontal eye field to the intraparietal cortex results in persistent hemispatial neglect.

In patients with hemispatial neglect, the direction of their gaze and their visual priority map are shifted relative to their proprioceptive reference frame (that is, the perceived location of their arm relative to the direction straight ahead). Balslev and Odoj (2019) asked patients to discriminate letters on a screen while looking at the location at which they felt their finger to be located behind the screen. This test was performed with the finger cue placed across a range of locations in the patient’s right visual field. To determine where each patient was looking relative to the position of their finger (from which they had only proprioceptive input), reaction time in the discrimination task was measured across a range of locations in the patient’s right visual field. In control participants (healthy elderly and right-brain-damaged patients who did not have hemispatial neglect), visual discrimination performance was the best at the location of the proprioceptive target (their hidden finger). In contrast, the patients’ focus of attention was shifted 0.5–2 degrees to the right at all tested cue locations across the right visual field.

Recall that damage to the intraparietal cortex disrupts saccade remapping in the double-step saccade task (Duhamel et al. 1992; Heide et al. 2004). So, in a patient with unilateral right parietal damage causing left hemineglect, as the patient’s eyes move to explore the visual scene, object locations can be correctly remapped after each eye movement toward the right but not after each leftward eye movement. For this reason, the locations of objects located contralesional to the current focus of attention will always be indexed as less reliable than objects ipsilesional to the current focus of attention. Since acting effectively on objects in an egocentric reference frame requires accurate information about the locations of objects, those objects whose locations are unreliable will be weighted as less salient when competing with objects whose locations are more reliably encoded, and they are assigned less weight in the generation of a salience map of indexed locations. Since the location of objects is not reliably encoded after leftward eye movements, the locations of objects viewed after a leftward saccade will not be remembered correctly. Patients with hemispatial neglect were shown to have impaired working memory for the location of contralesional objects as they performed a visual search task, with the result that they repeatedly rescanned objects that they had already looked at (Husain et al. 2001; Pisella et al. 2004).

### 9.2. Visual Processing Outside of Awareness in Hemispatial Neglect

If impaired remapping results in a degradation in the reliability of information about the locations of objects in part of a scene and causes them to be excluded from awareness, what, then, is the fate of information about neglected objects in a scene? Cumulative research has shown that patients with hemispatial neglect do indeed process information about objects in a visual scene outside their ken, providing converging evidence that the identity of “neglected” contralesional information is encoded outside of awareness. For example, extinguished stimuli produce semantic priming. If a picture of a baseball is flashed in a patient’s left visual field, even if it is not seen, the patient is faster to recognize a picture of a baseball bat presented straight ahead (Berti and Rizzolatti 1992; McGlinchey-Berroth et al. 1993). Geometric illusions, like the Judd and Müller-Lyer illusions, can also be generated without awareness of the stimuli inducing them (Olk et al. 2001; Ro and Rafal 1996), and neglected information is used in segregating the figure from the ground based on symmetry (Driver et al. 1993). When shown two objects, one of which is somehow defective on the contralesional side, patients may report them to be identical but still indicate a preference for the non-damaged object (Doricchi and Galati 2000; Marshall and Halligan 1988). Extinction is less for meaningful than for meaningless stimuli (Ward and Goodrich 1996), and it is reduced by emotionally salient stimuli; e.g., there is less extinction if the contralesional stimulus is a spider than if it is a flower (Vuilleumier and Schwartz 2001a). Extinction is also less likely if the contralesional stimulus is a face (Vuilleumier 2000), especially if the face appears angry (Vuilleumier and Schwartz 2001b) or if the contralesional stimulus matches the content of working memory (Soto and Humphreys 2006).

### 9.3. Visual Detection Is Gated by Attending to Action: Evidence from Hemispatial Neglect

**Figure 14:**
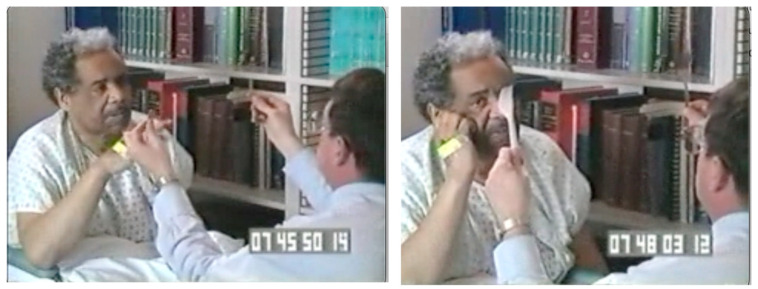
Extinction and stimulus repetition. Patients with visual extinction are more likely to demonstrate visual extinction if the two competing items are the same than if they are different; see text (from a video by Perpetua Productions).

Figure 14 illustrates that extinguished contralesional objects are, indeed, encoded pre-attentively. Instead of wiggling fingers, confrontation testing is being conducted by holding up objects in one or both visual fields and asking the patient, “what do you see”? Immediately before the two frames from the video (Video S6, “Feature dependent extinction video for Figure 14” from Perpetua Productions, provided in the Supplementary Materials) that are shown in the Figure 14, the examiner held up two identical coins (quarters), one in each visual field. The patient looked at, and pointed toward, the coin on his right and reported, “a quarter”, and he ignored the coin to his left. Next, as shown in the left frame of Figure 14, the examiner held up coin on the patient’s right and a key on his left. The patient looked at, and then pointed at, the coin on the right; he then looked at and pointed to the item on his left and reported seeing “a key” (Figure 14, left). Therefore, extinction was observed when the two competing objects were the same but not when they were different. The fact that extinction was modulated by the identity of the two competing object indicates that both their identities must have been processed—at least to the degree that permitted them to be encoded as being somehow different from one another.

The right image in Figure 14 shows the same test, conducted a few seconds later, in which two forks are presented. The patient detects and reports the fork to his right but ignores the fork to his left. Again, when the two items were the same, extinction was observed. But note that the two forks were not identical. They had different colors: a white plastic picnic fork was on the right, and a silver metal dinner fork was on the left. So, why did the difference in color not cause the fork on the left to be detected? The reason, it turned out, was because the patient was not asked to report the color of the objects. He was simply asked, “what do you see”? This question prompts a task tacitly based on shape.

In a subsequent experiment in this patient and four other patients with visual extinction, the patients were instructed to report either the shape or the color, in separate blocks, of colored letters briefly presented on a computer screen (Baylis et al. 1993). The stimulus duration was adjusted individually for each patient such that extinction occurred in half–two-thirds of the trials in which the colored letters were presented bilaterally. The patients were instructed to report the feature (color or letter) and the location of each item that they saw; if the patient only saw one colored letter on the right, they were to report the required feature of that item and to report seeing “nothing” on the left”. So, for example, in the test shown on the right of Figure 14, the correct response on a color report test would have been “white on the right and silver on the left; while in the shape-report test, the correct response would be “fork on the right and fork on the left”. If the patient reported “fork on the right and nothing on the left”, the outcome of that trial was designated extinction. That is, if the patient reported seeing something on the left but named the wrong feature, that trial was not scored as extinction. Trials were only scored as showing extinction when the patient explicitly denied detecting any contralesional stimulus.

The results showed that the presence or absence of extinction was determined by whether the objects were same or different in the dimension that the patients were instructed to report. If the patient was reporting the identity of the competing letters, there was more extinction if the letters were the same, but the colors of the items, whether the same or different, had no influence of the amount of extinction and vice versa; in blocks in which the patient was reporting the color of the competing letters, there was more extinction if the colors of the letters were the same but the identity of the letters, whether they were the same or different, had no influence of the amount of extinction.

Therefore, the patients’ access to awareness of the competing contralesional stimulus was modulated by whether the competing objects were same or different, but only if they differed in the task-relevant dimension. However, the following question remains: in what respect are the objects the same or different? Is it a matter of the same visual features (shape vs. color) or the same meaning (if both items are forks)? Or does it require that they provoke the same response (saying the word “fork” for the stimulus on the right and “fork” for the stimulus on the left)? Subsequent experiments showed that access to consciousness was gated by attending for action; i.e., extinction was more likely if the two items competed for the same response, even if their visual features were different or even if they had a different meaning (Rafal et al. 2002). Thus, there was as much extinction between ONE + 1 as there was between ONE + ONE or 1 + 1; and there was as much extinction between WON + ONE as there was between ONE + ONE.

While attention is often discussed as being necessary to allocate limited neural processing resources, the observations discussed in this section suggest that the constraints imposed by action systems are what limit accessibility to consciousness. After all, we can either turn left or right. We only have two arms, and even if we had eight, like the octopus, the actions that we can execute simultaneously are finite. As we move through the untraveled world, we must sequence our actions, and we are aware only of objects in a scene that are relevant for ongoing actions at any one instant. But, as we have also seen, information about other objects in the scene, although outside our ken, are fully processed by the visual system but without precise information about their location in egocentric coordinates. They are immediately available, but they are left unused until they are selected for action.

### 9.4. Recalibrating the Dorsal Stream: Prism Adaptation

Earlier, we saw that infants learn to use an efference copy of eye movement commands from the superior colliculus to remap the perceived locations of objects selected for action and that this learning of sensorimotor contingencies is achieved by reconciling conflicts between visual and proprioceptive information. The realignment of visual signals and proprioceptive signals enables visual information to be represented in body-centered (egocentric) coordinates, which are needed to guide action.

If visual information about the location of an object is perturbed, for example, by viewing an object through wedge prisms that refract light, and the viewer is asked to point toward the object, the individual initially reaches toward the perceived location of the object and misses, generating an error signal. This error signal generates complex spikes in cerebellar Purkinjie cells. These error signals are used to realign visual and proprioceptive information such that the observer adapts to the prism-induced visual shift and, after several misses, learns to reach accurately toward the target. Effective adaptation to the prismatic shift is demonstrated by showing that upon the removal of the wedge prisms, an after-effect is observed in which there is a pointing error is made in the opposite direction of the prismatic shift. That is, the patient again misses the target and has to de-adapt by again re-aligning their visual and proprioceptive reference frames.

**Figure 15:**
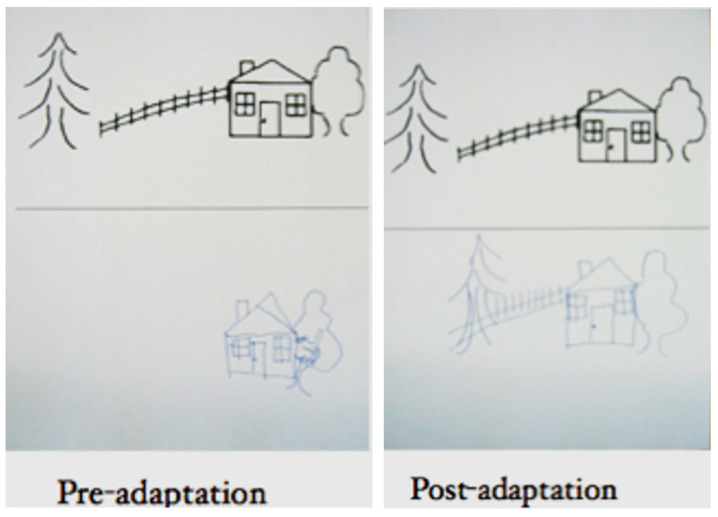
The patient was asked to copy the figure shown at the top of each panel. The patient’s copies are shown on the bottom of each panel before (**left**) and after (**right**) prism adaptation.

So, without making any further lesions in the brain, as can be performed in experimental animals to recalibrate the function of the dorsal stream (the Sprague effect—see below), the dorsal stream can be temporarily (for about two hours) and non-invasively recalibrated in humans by exposing patients with hemispatial neglect to prism adaptation. Figure 15 shows copies of a picture made by a professional artist who had severe hemispatial neglect before and after adaptation to right-refracting wedge prisms.

### 9.5. Recalibrating the Dorsal Stream: The Sprague Effect

The behavior of patients with unilateral damage to the parietal lobe reflects an imbalance between the two hemispheres of the brain that manifests both as a bias against orienting contralesionally and a bias toward orienting ipsilesionally. This opponency has been posited to reflect hemispheric rivalry in which each hemisphere inhibits the opposite hemisphere (Kinsbourne 1977). When one hemisphere is damaged, the behavioral consequences result not only from a loss of function in the damaged hemisphere but also from a decrease in the inhibition of the undamaged hemisphere. The disinhibited intact hemisphere then further inhibits the function of the damaged hemisphere (Blankenburg et al. 2008). Thus, the pathological disinhibition of the intact hemisphere contributes to hemispatial neglect via the further inhibition of the function of the damaged hemisphere.

Moreover, this hemispheric imbalance is operative, not just at the level of the cerebral hemispheres but throughout a network (see Figure 5) comprising both cortical and subcortical structures. In a seminal experiment, James Sprague (1966) induced contralesional hemispatial neglect in cats via the surgical ablation of the visual cortex in one hemisphere. He then showed that the ablation of the superior colliculus in the opposite hemisphere, contralateral to the cortical ablation, (or the transection of the collicular commissure connecting the two superior colliculi) (Wallace et al. 1990) restored orienting toward the neglected side. Further, Rushmore et al. (2006) showed that lesion-induced hemispatial neglect in cats is associated with hypoactivity of the ipsilateral superior colliculus and hyperactivity of the contralateral superior colliculus. Depressing the hyperactivity in the intact side by cooling the superior colliculus in the hemisphere contralateral to the cerebral lesion resulted in a restoration of orienting.

### 9.6. The Reverse Sprague Effect: The Role of the Frontal Eye Fields in Generating Voluntary Eye Movements and in Controlling Midbrain Eye Movement Reflexes

The Sprague effect is not caused by inhibitory projections from one superior colliculus to the other. Injections of ibotenic acid (which inactivates neurons but has no effect on the white matter fibers of passage) into the colliculus contralateral to a neglect-inducing lesion of the visual cortex does not produce the Sprague effect (Wallace et al. 1989). That is, inactivating collicular neurons but not the white matter fibers that pass through the colliculus does not ameliorate neglect. 

What, then, are the fibers which, when interrupted, produce the Sprague effect? Each superior colliculus receives input from the frontal eye fields that is relayed via the basal ganglia, specifically from neurons from the substanta nigra pars reticulata (Hikosaka and Wurtz 1983). These afferents from the basal ganglia inhibit the ipsilateral superior colliculus. Additionally, one part of the substantia nigra pars reticulata sends fibers of passage through the ipsilateral superior colliculus that cross the midline to inhibit the opposite superior colliculus. It is the interruption of these crossed inhibitory projections, which transmit neural signals from the frontal eye field of one hemisphere to the superior colliculus of the opposite hemisphere, that is responsible for the restoration of orienting seen in the Sprague effect.

The frontal eye fields generate commands to execute voluntary eye movements. These eye movement command signals from the frontal eye field are projected (mainly through the basal ganglia) to the deep motor layers of the superior colliculus. Damage to the frontal eye field in one hemisphere (Henik et al. 1994), or the neurodisruption of the frontal eye field with transcranial magnetic stimulation (Ro et al. 1997), results in an increase in latency to initiate a contralateral voluntary saccade to the contralateral visual field in response to a central arrow cue (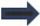). These signals from the cortex to initiate a voluntary saccade compete with external signals encoded in the salience map in the colliculus.

Acute lesions to one of the frontal eye fields often cause hemispatial neglect contralateral to the damaged cortex. However, neglect due to frontal eye field lesions typically recovers rapidly (within days or weeks of the injury). The transient hemispatial neglect observed in this circumstance reflects the disorganizing effects of perturbing the entire network (see Figure 5) of which the dorsal stream is a part—a phenomenon known as diaschisis. What is the neural mechanism that underpins the rapid recovery from hemispatial neglect after unilateral lesions of the frontal eye fields?

Although the transient inactivation of the frontal eye field of one hemisphere with transcranial magnetic stimulation impaired the generation of voluntary saccades toward the contralesional field, it had no effect on the latency to initiate saccades to exogenous visual targets in either direction. In contrast to the effects of transient frontal eye field neurodisruption in healthy volunteers, in patients with chronic unilateral lesions of the frontal eye field in whom signs of hemispatial neglect had long since recovered, the latency to initiate saccades to a visual target was increased; but it was increased toward visual signals presented in the ipsilesional (good) visual field (Henik et al. 1994). Although reflexive eye movements toward a suddenly appearing visual target are typically initiated with much shorter latencies than voluntary saccades are, in patients with chronic frontal eye field lesions, the latencies to initiate saccades toward an ipsilesional visual onset target were prolonged and were no shorter than the latencies to initiate voluntary ipsilesional saccades. These findings reveal that in patients with chronic lesions of the frontal eye field, eye movements toward the ipsilesional field are executed voluntarily without any reflexive contribution from the salience map in the superficial layers of the superior colliculus.

These observations suggest that recovery from hemispatial neglect after lesions affecting the frontal eye field is mediated by the inhibition of the superior colliculus in the intact hemisphere and, consequently, the disinhibition of the superior colliculus on the side of the frontal eye field lesion. Projections from the frontal eye field to the superior colliculus thus appear to play an important compensatory role in recovering from hemispatial neglect following damage to the frontal eye field. Disruptions to contralesional orienting responses at the cortical level are compensated by a rebalancing at the subcortical collicular level. This rebalancing biases against attentional capture and orienting toward the ipsilesional field and reduces signs of hemispatial neglect.

### 9.7. Cortical Control of Collicular Eye Movement Reflexes

Presumably, the crossed projection from the basal ganglia that exerts inhibitory control over the contralateral superior colliculus was not selected during evolution in order to help an injured organism recover from the effects of damage to the frontal eye fields. The ability of the frontal eye fields to exert control over reflexive orienting could, however, provide a mechanism for the frontal eye fields to orchestrate the integration of voluntary and reflexive eye movements. A neural signal sent from the frontal eye field to the ipsilateral superior colliculus to execute a voluntary eye movement would bias the colliculus to generate a saccade toward the intended target, while a crossed projection from the frontal eye field via the basal ganglia to the opposite colliculus would decrease the salience of competing visual external signals in the opposite colliculus.

There is evidence that people are able to exert top-down control over the collicular circuitry that mediates oculomotor reflexes. The visual grasp reflex, which was introduced when we discussed the symptoms of collicular degeneration in patients with progressive supranuclear palsy, consists of two opponent components. A visual stimulus suddenly presented in the peripheral visual field grasps the eyes and tugs them to move toward the location of the stimulus; it then locks the eyes on the stimulus, now at fixation, to lock the eyes on the target and to track it if it moves (a fixation reflex). Neurons in the rostral pole of the colliculus have visual receptive fields at fixation. After the visual grasp reflex summons the eyes to fixate a new visual target, those fixation neurons fire to initiate a fixation reflex that holds the eyes on the target and inhibits saccades elsewhere.

These opponent components of the visual grasp and fixation reflexes can be studied using a simple fixation offset paradigm. Participants are asked to fixate a plus sign (+) on the center of a computer screen and to make an eye movement toward visual targets that appear randomly on the left or right. In half of the trials, the fixation point is extinguished simultaneously with the onset of the peripheral visual target, and in the other half of the trials, the fixation point remains visible. In trials in which the fixation plus sign remains visible, the latency to initiate eye movement is longer in comparison to the fixation offset condition because the fixation point reflexively anchors the eyes at fixation, inhibits saccade-generating neurons, and delays the onset of the saccade to the peripheral target. The reduction in saccade latency in the fixation + offset condition is called the fixation offset effect (FOE) (in some variants of this paradigm, there is a temporal gap between the offset of the fixation plus and the onset of the peripheral target, and the difference in saccade latency in the two conditions is called the gap effect). This effect reflects mutual inhibition between fixation neurons (that are activated by the fixation + sign) in the rostral pole of the superior colliculus and more caudal movement neurons that are activated by the peripheral target stimulus. Offsetting the fixation point reduces the inhibition of movement neurons, resulting in a shorter latency to initiate the saccade toward the peripheral target (Dorris and Munoz 1995).

Machado and Rafal (2004) showed that people are able to voluntarily inhibit the fixation reflex and that this ability is under the control of the frontal eye fields. We showed that if participants are given a central arrow cue that predicts where a visual target will appear so that they can prepare an eye movement before the target’s onset, the fixation offset effect is reduced (Machado and Rafal 2004). Before the appearance of the peripheral target, participants were shown either an uninformative cue at the center of the display (<>) or an informative cue that predicted the location of the target (>) and enabled participants to prepare a saccade toward it. The cues at fixation either remained visible, or, with equal probability, were offset at the onset of the target. In healthy control participants, the latencies of saccades were reduced when the precue was informative; in addition, the size of the FOE was reduced in the informative precue condition, showing that when participants prepared a saccade, they were able to reduce the collicular fixation reflex prior to the appearance of the peripheral visual target. Patient with unilateral lesions of the frontal eye field also reduced the latencies of saccades to both the contralesional and ipsilesional targets. When patients executed saccades toward ipsilesional targets, the FOE was reduced. In contrast, the FOE was not reduced by an informative precue when saccades were made toward a contralesional visual target.

## 10. What Is a Scene?

The picture in Figure 15, which the patient was asked to copy, is itself a representation of a visual scene. The job of the dorsal stream is to enable the copier of the picture to re-represent the picture of the scene.

While we commonly think of a visual scene as what we see when our eyes are open, we can mentally represent a scene with our eyes closed—this is referred to as visual imagery. Try this. With your eyes closed, imagine the capital letter D. Now, in your mind’s eye, rotate the letter D so that it is lying on its side with the round side facing down. Now, also imagine the number four in your mind’s eye. Now, in your mind’s eye, put the number four right on top of the rotated letter D that you see in your mind’s eye as lying on its side with the round part facing down. What do you see now in your mind’s eye? Do you see a sailboat?

**Figure 16:**
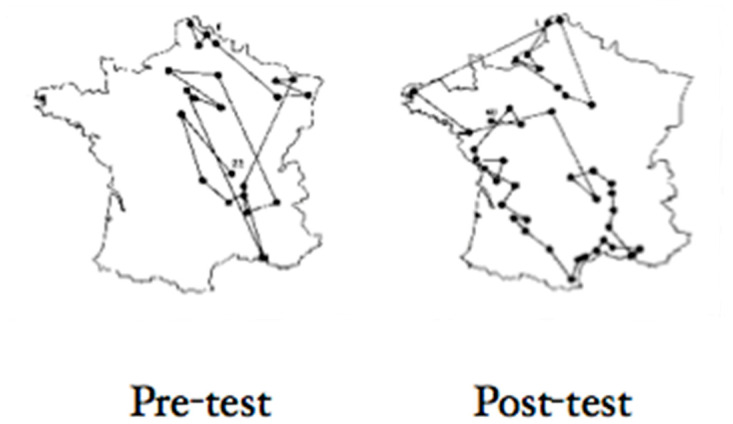
Representational neglect reduced after treatment with prism adaptation (adapted from Rode et al. 2001, Fig. 2, p. 1253).

Now close your eyes and imagine that you are looking at a map of your state or country and name all the major cities that you can see on the map in your mind’s eye. Figure 16 shows that hemispatial neglect manifests not only when viewing scenes perceived with the benefit of visual input but also when using visual imagery to view a scene in the mind’s eye. In this experiment (Rode et al. 2001), the patient was in hospital in Paris and was asked by the examiner to mentally visualize a map of France and report verbally all the major cities that the patient could “see” on the map. The scan paths shown in the two figures shown in Figure 16 depict the order in which the patient named the cities before (left) and after (right) prism adaptation. The figure on the left reveals that before prism adaptation, the patient neglected the contralesional left (west) side of the mentally viewed image of the map of France. The figure on the right shows that neglect improved after treatment with prism adaptation: the patient named more cities after treatment and reported cities on the west (the contralesional left side) of the mental representation of the map of France that had been neglected before prism adaptation.

Observations like those of Rode et al. (2001) demonstrate that hemispatial neglect affects mental representations of scenes seen in the mind’s eye. Their observations further demonstrate that the dorsal stream is involved in generating mental imagery and that the perturbation of dorsal stream function via the manipulation of sensorimotor contingencies can shift the patient’s mental visual search toward previously neglected parts of the mentally viewed scene.

### 10.1. A Word about Rehabilitation

Note, however, that after prism adaptation, the patient reported by Rode et al. (2001) failed to name cities on the right (East) side of the mental map of France which she had just “seen” and reported before prism adaptation. This observation suggests that prism adaptation does not “cancel” neglect. Rather, the manipulation of sensorimotor contingencies with prism adaptation shifts the bias in determining which parts of the mental image of the map is scanned by the mind’s eye. That is, prism adaptation does not cure hemispatial neglect. It may perturb sensorimotor transformations to transiently recalibrate the focus of attention contralesionally, but it does not compensate for a declining gradient of attentional resources from right to left. Patients with hemispatial neglect are impaired in visual searching, even in the vertical plane within the ipsilesional visual field (Vuilleumier and Rafal 2000). So, the temporary leftward shift in orienting induced by prism adaptation ameliorates only one component of the patient’s impairment.

The goal of this tutorial is to highlight clinical observations that provide insight into the origin and function of the dorsal visual stream and to consider how these insights can help clinicians better understand the pathophysiology of disorders like Balint’s syndrome and hemispatial neglect. Obviously, though, the goal of understanding the pathophysiology of any medical disorder is to guide the search for an effective therapy that can improve patients’ quality of life. As a clinician or a patient, the question to be posed of a potential therapeutic intervention is not whether “it works” but “does it help?” On this score, progress has been disappointing.

Prism adaptation certainly works insofar as it is routinely found to temporarily improve performance on some bedside tasks of neglect in some patients. Moreover, Saj et al. (2013) have shown that improvement in task performance in patients with hemispatial neglect was accompanied by increases in fMRI activations of the parietal and frontal regions of the dorsal stream bilaterally. However, prism adaptation does not improve visual searches for an item in an array of distracters (think *Where’s Waldo*) (Morris et al. 2004). Visual searches precisely represent the problem confounding patients with hemineglect: “Where did I put my glasses”? An inclusive meta-analysis of clinical trials which used a cancellation task (in which, for example, patients were asked to cross out all O’s scattered in a field of Q’s on a piece of paper) as a search task, revealed no benefit of prism adaptation (Székely et al. 2023).

Can the hemispheric rivalry principle revealed by the Sprague effect be harnessed to fashion a therapeutic intervention? While lesioning the left superior colliculus in patients with left hemineglect is obviously not an option, Michael Posner and I (Posner and Rafal 1987) suggested that it might be possible to achieve the benefits of the Sprague effect non-invasively by simply patching the right eye. As we have seen in patients with left hemineglect, there is an orienting bias such that orienting toward visual signals in the contralesional left visual field is reduced, and this bias against orienting contralesionally is accompanied by disinhibited hyper-orienting toward visual signals on the right. Patients with their right eye patched can still see visual signals in their right visual field, of course. However, since visual signals in the right visual field are transmitted to the left superior colliculus mostly from the right eye, we reasoned that patching the right eye could reduce left collicular activation and thereby reduce the resulting reflexive tug pulling the eyes to the right. Of course, seeing is easier with two eyes than with one, so even if eye patching is effective in reducing reflexive orienting to distracters on the right, it was not clear whether patients (or therapists) would find that the benefits outweighed the costs.

Butter and Kirsch (1992) reported improvements from monocular eye patching in patients with neglect, and Schenke et al. (2023) confirmed that monocular eye patching modulates ipsilesional reflexive saccades in patients with left hemispatial neglect. However, a four-week blinded clinical trial in an occupational therapy clinic (Tsang et al. 2009) that compared eye patching with conventional occupational therapy treatment found that although patients who were treated with eye patching showed more improvement on a standard battery of pencil and paper tests than did patients in the control group, at the end of the four-week trial, there was no difference between treatment groups in functional independence measures.

### 10.2. Seeing Is a Verb: The Scene That We See Is a Representation That We Continuously Reconstruct to Be Used on the Fly for Controlling Our Actions

When we view the world around us, we use the world as an “external memory” (O’Regan and Noë 2001) to generate a representation of a scene. That is, what we see is not just what our retinae harvest as they scan the scene before us. We use our memories of past scenes as well. Since we mastered, as infants, the visuomotor skills needed to reconstruct a representation of a visual scene, we have learned a lot about scenes throughout our lives, and we use what we have learned about scenes in reconstructing the representations that we experience as seeing.

Proceeding from observations on the robust phenomenon of boundary extension, Intraub (2010) has advanced a multisource model that conceives of scene perception as an act of spatial cognition that is not necessarily tied only to the visual modality. O’Regan (1992) proposed that seeing “is the action of interrogating the environment by altering retinal sensations, and by integrating these sensations into one’s cognitive framework” (O’Regan 1992, p. 475). On this account, computations in the dorsal stream construct a spatial scaffold of indexed locations in egocentric co-ordinates, selected for their current salience, that is available to be populated with objects for incorporation into the representation of the perceived visual scene. Intraub (2010, p. 235) argues that this egocentric spatial framework “serves as the ‘’bones’ of scene perception, that are then ‘fleshed out’, … by visual representation, amodal perception, and associations to both general and specific contexts”.

This framework for understanding scene perception can perhaps be best illustrated by considering what happens to viewed scenes when we attempt to scan them when our eye movements are paralyzed (Whitham et al. 2011). Paralysis was achieved using a drug (used in anesthesia) that paralyzes muscles but that has no effect on the nervous system. When volunteers under complete paralysis make eye movements while viewing a spot of light in a dark room, what they see is what you would expect. When attempting to execute an eye movement to the right, away from the target light spot, an efference copy of the eye movement command is transmitted from the superior colliculus to neurons in the dorsal stream. This efference copy transmits a prediction that the image will translate across the retina (from right to left), but because the eye muscles are paralyzed so that no translation across the retina actually occurs, the viewer “sees” the light spot jump to the right. Some subjects who made spiral eye movements or who moved their eyes to form shapes reported seeing the shapes that they drew with their eyes.

In this case, the participants were viewing a simple scene consisting of an isolated light spot in the dark. There were no other objects visible that could anchor the light spot in the external environment. Critically, though, this jumping of an object in a visual scene did not occur when the paralyzed subjects moved their eyes while viewing a scene in a lighted room which afforded a view of a full and visually complex scene. In this experiment, two experimenters (whose faces were known to the participants) stood, one on each side, of the paralyzed individual. The participants were instructed to alternate looking back and forth between the faces of the two experimenters. In this task, the paralyzed participants attempted to make voluntary eye movements. This effort would have resulted in activating the frontal eye field, which would have sent the motor command to the superior colliculus, and the colliculus would have sent a command to execute an eye movement. Although no saccade was made because the muscles had been paralyzed, the efference copy of the motor command would nevertheless have been recurrently sent to the cortex to remap the expected location of the fovea.

In this situation of viewing a natural scene, however, the participants did not report that the whole scene jumped back and forth as they moved their eyes back and forth. They used their knowledge about visual scenes, which they had acquired in infancy (including the fact that the world does not jump when we look around a scene before us), to stabilize the perceived scene, and they did not experience the objects in the scene jumping. In fact, four of the six participants experienced an illusion of clarity—i.e., they reported seeing the face toward which they had attempted to look clearly, even though the face was still in their peripheral vision. That is, those participants saw what they expected to see. Our brains fill in missing sensory information to afford us the experience of a complete and clear scene.

This illusion of clarity may seem bizarre; but perhaps it should not. When we look at the scene before us, we do not perceive objects that we are fixating as being clear while seeing everything else as becoming progressively blurrier in the visual periphery, from which only low-resolution visual information is actually available because the density of cone photoreceptors progressively declines across the retina.

There are several mechanisms that are likely at play in generating the illusion of clarity and perceptual continuity (Marino and Mazer 2016). As mentioned earlier, since infancy, we gain a lot of experience viewing scenes, and we have never had the experience of everything in a scene jumping at once. On this account, seeing is an act of cognition in which we use the world as an external memory. At any instant, our retinae provide us with the gist of a scene and “The subjective impression we have of seeing whole objects arises, first, because the retinal stimulation is very rich and so provides the impression that ‘a lot of stuff is out there’, and second, because if at any moment we want to know what exactly any of the stuff is, we just use our retinas and eye movement to find out” (O’Regan 1992, p. 474) … “It is the act of looking that makes things visible” (p. 475). However, since the faces that the paralyzed participants were attempting to look at did not land on the high-acuity fovea, the experience of clarity was an illusion, and a control reading experiment confirmed that there was no actual improvement in visual acuity.

Another explanation for the illusory experience of clarity is that an activation of the frontal eye field by an attempt to generate an eye movement generates a shift in attention to the location of the attempted saccade location. Moore and Fallah (2004) showed that even low intensity electrical stimulation of the frontal eye field, that was below the threshold needed to generate a saccade, elicits a shift in attention in monkeys. Further, temporary inactivation of monkey frontal eye fields impaired covert shift of attention (Wardak et al. 2006). Moreover, when an eye movement is made toward an object, the sensitivity to higher spatial frequencies highlighting the details of the object is enhanced even before the eye movement begins (Kroell and Rolfs 2021).

Marino and Mazer (2018) showed in monkeys that eye movement signals mediated a “presaccadic transfer of attentional state” in the extrastriate cortex (area V4 in their experiment) that may be responsible for the beneficial effect of covert attention—an ability to shift attention while maintaining steady fixation—that evolved in primates and other social and prey species. This ability enables a non-dominant animal to keep track of the actions of the alpha male without attracting attention, or it enables a prey animal to keep track of a predator without establishing eye contact that could attract the predator’s attention (Emery 2000).

By O’Regan’s (1992) account, then, the dorsal visual stream contributes to the awareness of only a limited number of items, and these selected items are plotted, in egocentric coordinates, on a perceptual canvas that provides the gist of a scene that is available as an external memory: “Because the image is continuously available out there … the task of vision is to extract just a sufficient number of cues from this external memory store, so that objects can be discriminated from one another and so that manipulation of objects and locomotion become possible (p. 480)”.

As we have seen, the index of locations from which objects are selected for action is actually quite sparse. In fact, we can fail to report seeing an object right in front of us that is plotted on our foveae if that object is not relevant to the task at hand (Mack and Rock 1998). Therefore, seeing “does not involve simultaneously perceiving all the features of an object, but only a very small number, just sufficient to accomplish the task at hand” (O’Regan 1992, p. 481).

The gist of a scene is generated by the computation of summary statistics in structures of the ventral stream (such as the parahippocampal gyrus and retrosplenial cortex) that encode visual ensembles (Cohen et al. 2016). These computations of summary visual statistics enable us, in the “briefest of glances” (Greene and Oliva 2009), i.e., without time to make any eye movements, to classify a scene as a beach, desert, mountain range, forest, crowd of people or animals, etc., to provide context about the current environment (e.g., safe or dangerous, indoor/outdoor, season of the year) and potential constraints (such as openness and navigability). The context and constraints imposed by the perceived gist of the scene biases the assignment of the salience of objects indexed in the scaffolding generated by the dorsal stream and “not only gives observers and immediate sense of the visual world, but also provides a foundation for further exploration” (Cohen et al. 2016, p. 329).

Inattentional blindness, manifested as change blindness (Simons and Ambinder 2005), was first demonstrated in an experiment conducted by Neisser and Becklen (1975). The main purpose of their experiment was to investigate whether information in one visual channel could be attended while a second channel is ignored, analogous to the cocktail party effect that had been previously observed in the auditory modality (if you are listening to a conversation at a party, you cannot simultaneously listen to a different conversation). They projected two different videos depicting people playing a game. In one video scenario, three men were passing a basketball, and participants were tasked with counting the number of passes. In the other scenario, two men were playing a hand-slap game. Performance was measured in each task under three conditions: in one condition, each video was shown to participants separately. In the two other conditions, both videos were projected superimposed upon one another on a screen, and subjects were tasked with attending to either the ball game or the hand-slap game; in a third condition, they were tasked with attending to both games and reporting the score in each (most readers will have experienced this situation when looking out of a window under the right lighting conditions such that both the scene outside the window and the reflection of the room behind the viewer are visible; you can “see” one scene or the other, but you cannot attend to both at the same time). As expected, the participants attempting to perform both tasks simultaneously performed abysmally.

The investigators also intercalated, in each of the two scenarios, “unusual events”. For example, in the middle of the video of the hand-slap game, the two players briefly shook hands and then resumed playing. In the ball game, the three men playing at the beginning of the game were sequentially replaced by women players by the end of the video. Most participants tasked with monitoring the hand-slap task never noticed that the men passing the ball had been replaced by women. Most participants tasked with monitoring the ball game failed to notice the hand-slap players shaking hands.

The main question addressed in this experiment was whether selective looking requires the active, effortful suppression of the unattended visual channel. The results showed that participants required little if any effort in attending to one visual scene while ignoring another. For most participants, performance in monitoring one of two superimposed scenes was no worse than when performing the task when viewing one scene alone. The authors concluded that “Selective looking … while full and efficient, does not involve anything like ‘suppression’ … The perceiver picks up certain information … about an event or object, and uses it to construct a particular representation … Other information is simply left unused”.

## 11. Duplex Seeing: Seeing without a Scene Reconsidered

We have seen that bilateral damage to the dorsal visual stream causes the perception of a visual scene to constrict to awareness to just a single object that happens to fall randomly on the fovea as the eyes wander chaotically (a sign referred to as ocular apraxia). But is a single object all that a patient with Bálint’s syndrome sees when viewing a scene? Recall that the patient shown in Figure 3 was only able to report either the comb or the spoon but that at one point, both objects disappeared, and his eyes were captured by some chalk marks on a blackboard behind the examiner. What he reported, though, as not just chalk marks but rather “writing on a blackboard”. So, although patients with Bálint’s syndrome may have conscious access to the presence of only one object in the scene, they seem to see more than just a single object.

Moreover, although these patients do not seem to have conscious access to information about the locations of objects that they do see, there is nevertheless evidence that information about the locations of objects is encoded outside of their awareness. Kanwisher (1998) used a modified Stroop paradigm in which the patient was shown two words, one of which was colored, and asked to report the color that they saw and ignore the words. Although he was not able to report, explicitly, which word was colored, there was nevertheless a larger Stroop interference effect (i.e., he was slower to name the color) when the colored word name was incongruent with name of the color (e.g., blue) than if the uncolored word name was incompatible with the name of the color. Thus, there was implicit evidence that the word and its color had been bound, even though the patient had no explicit access to the conjunction of features.

The objects that are encoded in egocentric coordinates by the dorsal stream are not all that we see when we view a scene. The dorsal stream only represents the spatial positions of a few objects in relation to each other and to the observer, and only those objects that are currently candidates for directed actions that require precise spatial information in egocentric coordinates. Nevertheless, these objects are plotted on a canvas that affords us the experience of a full and coherent scene. Recall that even with very brief exposures that do not permit any eye movements, we are able to extract scene statistics that provide information about the gist of a scene (e.g., open and navigable, inside or outside, a beach scene, a mountain range, a forest) (Greene and Oliva 2009). Experiment, similar to those of Greene and Oliva (2009) have not yet been conducted in patients with Bálint’s syndrome. But, as we saw, the patient shown in Figure 3 reported seeing a scene consisting of a blackboard with some writing on it—not a free-floating white line in an otherwise empty world. Moreover, patients do not seem to be aware that anything is missing from the scene. They only discover what they do not see when they fail in attempts to interact with objects in their environment.

What we see when we view a scene, then, is a synthesis of visual information from both the dorsal and ventral streams. While the dorsal stream utilizes information predominantly from magnocellular input and the ventral stream utilizes predominantly parvocellular input, it is not the case that the dorsal stream only receives magnocellular signals and that the ventral stream only receives parvocellular signals. There is convergence of magno- and parvocellular pathways as early as the primary visual cortex in layer 4b (Sawatari and Callaway 1996). Moreover, neurons in the lateral intraparietal cortex do respond selectively to different shapes (Sereno et al. 2018).

It makes sense that the dorsal stream must have access to shape information. In primates, the dorsal stream must control not only orienting movements of the eyes, head, and trunk but also reaching and grasping movements that require accurate information about the size and shape of the object to be grasped. Bruner (2023) has highlighted the point that in humans, the function of the dorsal visual stream evolved to enable the development of new skills like throwing and tool use. Throwing, a skill not possible in non-human apes, was made possible by changes in the anatomy and mechanics of the shoulder that enabled modern humans to project force (Roach et al. 2013). When using tools, an analysis of ocular scan paths revealed that the gaze is first drawn to the functionally useful part of the tool, e.g., the handle, rather than to the most visually salient (e.g., the brightest) feature of the tool. That is, when using tools, voluntary orienting overrides exogenous orienting.

An experiment in patients with hemispatial neglect (di Pellegrino et al. 2005) showed that features of viewed objects that activate actions associated with those features—object affordances—automatically capture attention. Pictures of a teacup were presented in the left visual field, the right visual field, or both fields simultaneously. Patients made no judgements about the teacups; they simply performed a standard test for extinction in which they had to report, verbally, if they saw anything appear in their left field, their right field, or both fields. The teacups (in some trials) had handles on them, and, in these trials, the handles could be oriented either toward or away from the observer. All three patients had less extinction when the teacup in their left visual field had its handle oriented toward the patient (such that it afforded a grasping action) than when the handle was pointing away from them.

Although both what and where types of information are available in both ventral and dorsal visual streams, the two pathways nevertheless differ fundamentally in their roles in visual information processing. Yet each pathway utilizes information transmitted from the other visual stream for its own purposes. For example, the degree to which shape information is utilized in dorsal visual stream processing is dependent upon whether the shape information is task-relevant (Vaziri-Pashkam and Xu 2017).

Duplex seeing that integrates visual information from the dorsal and ventral visual streams thus contains two types of visual information:

- Dorsal stream information, mainly magnocellular, about the relative locations of a few task-relevant objects that is updated with each movement;
- Ventral stream information (mainly parvocellular) about the visual statistics of the background and the fine details (color, texture, and shape) of fixated items which does not need to be remapped after each eye movement.

**Figure 17:**
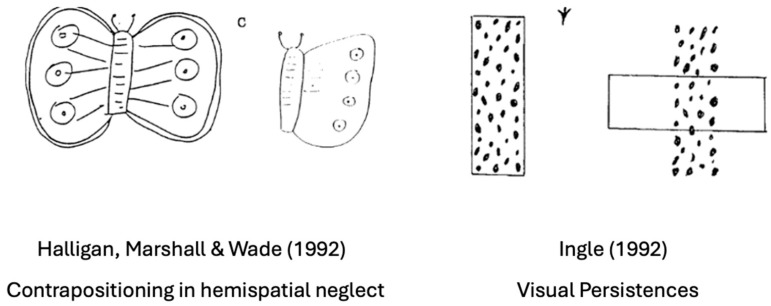
Duplex seeing: a double dissociation (see text). (**Left**): Adapted from Halligan et al. (1992, Fig. 1, p. 127). (**Right**): Adapted from Ingle (2004b, Fig. 7, p. 38).

Figure 17 depicts a double dissociation that dramatically illustrates duplex seeing. The figure on the left (Halligan et al. 1992, Fig. 1, p. 127) shows a copy of a butterfly drawn by a patient with left hemispatial neglect. It demonstrates how the deficit in remapping the locations of objects and their features after eye movement contributes to the impaired performance of patients with this disorder. Note the contrapositioning of several details. When first viewing the model, the rightward spatial bias draws the patient’s gaze to the right sides of the model, and the eyes must move toward the left (contralesionally) to copy the body, both antennae, and some horizontal lines depicting segmentation of the butterfly’s body. The patient’s eyes would then move rightward to draw the right wing. Note that due to the failure to remap after the leftward eye movement, there is a mislocalization and contrapositioning of some of the fine details in the figure relative to the global butterfly: the horizonal lines on the insect’s body are reduplicated to the right of their original position; also note the transposition of one ring on the left wing of the butterfly to the right wing.

Pisella and Mattingley (2004) have elucidated how the failure to remap after contralesional (leftward) eye movements accounts for some of the cardinal signs of hemispatial neglect and visual extinction. Consider the conventional bedside confrontation test for visual extinction (e.g., Figure 13). When the examiner raises both arms to start the test, the patient spontaneously makes an eye movement toward the examiner’s left hand in the patient’s right visual field. To begin the test, the examiner instructs the patient to “look at me”, and the patient then executes a saccade to the left to fixate the examiner’s face. However, this leftward saccade is not remapped and, therefore, the locations of the two competing stimuli (the wiggling fingers) are not veridically encoded. The resultant contrapositioning of the left stimulus toward the right does not permit the registration of the stimulus in the left visual field to be encoded as a distinct percept at a distinct location. Contrapostioning thus yields a percept of a single object at a single location in the right visual field, resulting in extinction from awareness that there is also a second stimulus in the left visual field.

Now, if the two objects have different shapes, information transmitted from the ventral stream to the dorsal stream provides information (if the shape is task-relevant) that distinguishes between the two objects and permits them to be registered as being in different locations from one another (e.g., Figure 14). Mislocalization errors in patients with parietal lobe damage, in which a stimulus on the left is mislocalized to the location of a stimulus on the right (a sign referred to as allochiria), can affect not just the location of visual stimuli but also the perceived location of tactile sensations and the locations of body parts. It has been proposed that allochiria may also explain bizarre reports from patients in which they deny that the left side of their body is paralyzed (anosognosia) or that they state that their left arm does not belong to them (somatoparphrenia) (Marcel et al. 2004).

Now, take a look at the second image shown on the right in Figure 17. The work of science is, as Plato tells us, to carve nature at its joints. David Ingle showed that he could, literally and personally, pull apart the magnocellular and parvocellular components of a scene with his bare hands. He discovered the rare phenomenon of visual persistences and reported systematic observations of the phenomenon in himself and four other individuals (Ingle 2004a, 2004b) He described these visual persistences as follows:

“… positive afterimages following brief fixation of high-contrast objects or drawings and eye closure. [Visual persistences] duplicate shapes and colors of single objects, lasting for about 15 s. Unlike retinal afterimages, [visual persistences] do not move with the eyes but are stable in extrapersonal space during head or body rotations. [Visual persistences] may reflect sustained neural activity in neurons of association cortex, which mediate object perception”.

(Ingle 2004a, p. 1135)

One example of a visual persistence is shown on the right of Figure 17 (Ingle 2004b, Fig. 18, p. 1320). Participants viewed a card held in their hand on which a rectangle constructed of dots bounded by a solid rectangle was drawn, as shown on the left. While still holding the card on which the figure was drawn, they then closed their eyes and saw that stimulus as a visual persistence. They then rotated the hand holding the card 90 degrees. In three participants, the group of dots was seen to rotate 90 degrees, while the solid rectangle bounding them did not rotate. In the other two participants, the dots faded quickly when the hand was rotated, while the solid bounding rectangle persisted. In contrast with the mislocalization errors made by the patient with neglect shown on the left of Figure 17, in which the positions of local details (processed in the ventral visual stream) migrated, the opposite was observed with the visual persistences shown on the right of Figure 17—here, the fine details migrated.

How is information from the ventral and dorsal visual streams integrated to provide us with the experience of a full and coherent scene before us? Damage to either the ventral premotor cortex or the temporo-parietal cortex (the junction between the posterior part of the superior temporal gyrus and the parietal lobe) can also cause the syndrome of hemispatial neglect. These regions have been identified as parts of a ventral attention network that works in concert with the dorsal attention network (the intraparietal cortex and the frontal eye fields) to regulate spatial attention (Corbetta and Shulman 2011). The scenes that we see presumably result from the interaction between the two attention networks. For example, the execution of eye movements triggers a predictive updating of attentional topography in the ventral stream (area V4), (Marino and Mazer 2018). Moreover, a temporary reduction in left hemineglect after adapting to right refracting prisms is associated with a shift in activation from the right temporo-parietal junction of the ventral attention network to the left tempo-parietal junction (Clarke and Crottaz-Herbette 2016).

Ingle (2004b) speculated that the prefrontal cortex plays a role in integrating the outputs of the ventral and dorsal visual streams. However, the temporo-parietal junction, a multisensory association cortex that is located at the anatomical intersection of somatosensory, visual, and auditory association cortices, is optimally located anatomically to integrate information from both magnocellular and parvocellular channels.

## 12. Summing up and Looking Forward

This tutorial has endeavored to review what we have learned about the origin and function of the dorsal visual stream from bedside observations of neurological patients and from laboratory experiments motivated by those clinical observations. It has considered the effects on visually guided behavior and on scene perception of damage to the superior colliculi or damage to the intraparietal cortex or the frontal eye field—two of the main targets of dorsal visual stream projections from the primary visual cortex.

Phylogenetically and functionally, the superior colliculus is the origin of the dorsal stream and remains its hub. In concert with magnocellular projections to the visual cortex, the colliculi transmit visual signals to the dorsal stream. The colliculi also project copies of eye movement signals to the dorsal visual stream that provide information about both the position of the eyes in the orbits and about which object in a scene has won the competition for attention. These two sources of information are integrated in the intraparietal cortex to reconstruct the scaffolding of a visual scene by indexing the locations of objects that have been selected for action and for access to consciousness.

Through monosynaptic projections from the retina, the superficial layers of the colliculi receive immediate warning signals of changes in luminance or motion in the scene. If a change in intensity, size, or movement is detected that signals a potential threat, these signals trigger an automatic orienting response toward the potential threat and also transmit an early warning alert, via a single synapse in the thalamus, to the amygdala. The colliculi also send motion signals via a single synapse in the inferior pulvinar nucleus of the thalamus to the motion–direction-sensitive cortical area V5/MT of the dorsal stream, bypassing the primary visual cortex (Berman and Wurtz 2010).

Thus, the superficial layers of the colliculus maintain a continuous salience map of signals currently impinging on the retina. This background collicular salience map is continuously modified by input from the primary visual cortex. The collicular salience map enables a fast and efficient search for visually salient targets. For example, if you are looking for a red book on a bookshelf holding all green books, the red book pops out. It does not matter how many green books are on the bookshelf; the salience of the singleton color will automatically summon your gaze to the red book.

For patients with degeneration of the superior colliculus, visual features do not pop out. Their visual search efficiency does not benefit from the detection of visual salience (Smith and Archibald 2018). Therefore, these patients have great difficulty finding things in their daily lives. Since the degeneration of the colliculus also deprives the intraparietal cortex of efference copies of eye movement commands, the patients are deprived of the information needed to encode eye position in the orbit. As a result, patients do not accurately encode the locations of objects, resulting in an impaired spatial working memory (Smith et al. 2021).

Bilateral damage to the caudal intraparietal cortex results in Bálint syndrome. Patients with this disorder have no conscious access to information about the spatial relationships of objects in the visual scene with each other or with their own body. As a result, patients are aware of only one object at a time—an object that is located wherever they happen to be looking at any one instant as their eyes wander. They are spatially disoriented, unable to use vision to navigate the environment or to reach and grasp the single object that they can see.

This is not to say that the patients with Bálint syndrome have no access to any information about spatial relationships. They have no problem, for example, in recognizing shapes and objects, and shape recognition requires spatial information about the relative locations of object parts. Information in the retinotopic maps of the primary visual cortex and the extrastriate cortex of the ventral visual stream is sufficient to permit object identification. Moreover, patients’ acquired knowledge of objects enables them to decode the identity of objects by integrating the relative spatial positions of their parts. For example, if a patient with Bálint syndrome is shown a display containing the following:

0 0

the patient will report seeing one

0.

If, however, the patient is shown a display containing two zeros connected by a solid line

0^------^0

the patient is more likely to report seeing a dumbbell or a pair of glasses (Humphreys 1998).

If a patient with Bálint syndrome is shown a picture of an American flag, the patient will most likely report seeing “a flag “rather than “a stripe” or “a star”. Many objects, like a flag, are hierarchical stimuli with a global level and local details. So, for example, houses have windows and doors, doors have door handles, and door handles may have key holes. In the examples of a flag or a house, each level in the hierarchy contextualizes the local details it that contribute to what is perceived.

Now let us consider an (artificial) situation in which the global and local levels of a hierarchical stimulus are unrelated and in which they provide no context that could influence the perception of the other level. In this video [Video S7 Balint Navon Video (Supplementary Materials) here], Lynn Robertson is showing hierarchical stimuli devised by David Navon (Navon 1977) to a patient with Bálint syndrome. The stimuli consist of either a large capital “A” constructed of smaller capital “S”s, or of capital “S”s constructed of smaller capital “A”s. The patient is being asked simply to “tell me what you see”! You will notice in the video that these Navon figures vary from very large to very small. You will also notice that regardless of the overall size of the stimuli, the patient reported only the local element and never reported seeing the global letter. This finding was consistently observed when the patient was tested on multiple occasions over several months. This video demonstrates that, as shown in Figure 3, the patient’s attention tends to be captured by fine details. In the case shown in Figure 3, the patient’s attention was captured by a linear chalk mark. We also noted, however, that the chalk mark was seen on the background of a blackboard; so, more was being seen than just an isolated white line.

If the attention of patients with Bálint syndrome is captured by single fine details, how can the perception of these details be modified by the context of the global configuration of a hierarchical stimulus like a flag or a house? Why is the patient likely to see a flag and not just one star or one stripe? It tuns out that although patients with Bálint syndrome are not explicitly aware of visual information at the global level of hierarchical Navon stimuli, it does not mean that this global information is not being processed or that it is not available to influence how visual information at the local level is perceived.

Karnath et al. (2000) conducted an experiment in a patient with Bálint syndrome in which the reaction time to identify a letter at the local level of hierarchical Navon figures similar to those shown in the video was measured. However in each trial, the global level, which the patient was not instructed to report, could either be compatible with the local level (i.e., coding for the same response) or incompatible with the local level (i.e., coding for the opposite response to that required by a correct response to the local level). The results reported longer reaction times to identify the local stimulus if the global stimulus coded for a response that was incompatible with the correct response to the local level. So, although the patient was not aware of the global stimulus, it was nevertheless processed and influenced the response to the local stimulus. We also saw earlier that the features of objects that patients are not aware of are processed outside of awareness and that these features can result in illusory conductions of features. Together, these kinds of observations indicate that while damage to or the dysfunction of the dorsal visual stream may prevent conscious access to more than one object in a scene, other elements of the scene are, nevertheless, processed outside of awareness and can influence what the patient does see.

This tutorial ends by considering how two channels of visual information, derived from processing in the dorsal and ventral visual streams, are integrated to reconstruct a coherent visual scene. Just as seeing with our cyclopean eye requires fusing the information from our two eyes, duplex seeing requires the fusion of the information processed in the ventral and dorsal visual streams. So, I will end by asking the following question: although the destruction of the dorsal visual stream causes all objects in a scene to disappear except for one object plotted on the fovea, what else does the patient see? Can a patient with Bálint’s syndrome discriminate whether a picture of a scene depicts a forest, a beach, a desert, or a meadow?

I do not know, but I would expect that they could. Our eyes dart about around three times a second, continuously sampling the visual environment. This is true of patients with Bálint’s syndrome as well. In fact, their darting eye movements are especially chaotic, particularly soon after the onset of the syndrome (a sign called ocular apraxia). Although patients do not know where their eyes are looking and do not perceive the locations of features in the scene that their eyes land on, they nevertheless receive statistical information about the visual properties comprising the gist of the scene. Therefore, a patient can use that information, and his lifetime of experience with viewing scenes, to infer, for example, that the white line his eyes have landed on is a chalk mark on a blackboard.

The study of neuropsychology can help us answer important questions about the brain and mind; but perhaps its greatest contribution is in telling us what the important questions are. We have yet more to learn from observations in the clinic and from just asking patients, “what do you see”? And from then taking those observations, and the questions they evoke, into the laboratory.

## Data Availability

No new data were created or analyzed in this study. Data sharing is not applicable to this article.

## Supplementary Materials

The following supporting information can be downloaded at: https://www.mdpi.com/article/10.3390/jintelligence12050050/s1, Video S1: Balint Eye movement for Figure 2, Video S2: Balint Chalk for Figure 3, Video S3: Social neglect. with Fig. mov, Video S4: $ neglect, Video S5: Axis-based extinction, Video S6: Feature dependent extinction, Video S7: Balint_Navon Video.
